# Perturbations in gut microbiota in autism spectrum disorder: a systematic review

**DOI:** 10.3389/fnins.2025.1448478

**Published:** 2025-05-16

**Authors:** Xiangkun Tao, Zhuocan Li, Dongfang Wang, Juncai Pu, Yiyun Liu, Siwen Gui, Xiaogang Zhong, Dan Yang, Haipeng Zhou, Wei Tao, Weiyi Chen, Xiaopeng Chen, Yue Chen, Xiang Chen, Peng Xie

**Affiliations:** ^1^Department of Neurology, NHC Key Laboratory of Diagnosis and Treatment on Brain Functional Diseases, The First Affiliated Hospital of Chongqing Medical University, Chongqing, China; ^2^Jinfeng Laboratory, Chongqing, China; ^3^Chongqing Institute for Brain and Intelligence, Chongqing, China

**Keywords:** autism spectrum disorder, gut microbiota, microbial biomarkers, microbial diversity, gut-brain axis

## Abstract

**Background:**

Autism spectrum disorder (ASD) is a neurological and developmental disorder commonly accompanied by gut dysbiosis and gastrointestinal symptoms. Accumulating evidence supports a crucial role of gut microbiota dysbiosis in the pathophysiological mechanisms of ASD. However, the alteration of gut microbiota shows high heterogeneity across different studies. This study aims to identify potential biomarkers in the gut microbiota of patients with ASD.

**Methods:**

We conducted a comprehensive analysis by searching the PubMed, Web of Science, Cochrane Library, and Embase databases, for studies assessing the changes of gut microbial diversity and taxa in ASD patients and healthy controls using high-throughput sequencing. Vote counting analyses were performed to identify consistently altered gut microbes associated with ASD.

**Results:**

Sixty-four studies involving 189 differentially abundant gut microbial taxa were included. Our synthesis provided no strong evidence for a difference in α-diversity between ASD patients and healthy controls, while studies were relatively consistent in reporting differences in β-diversity. Among 189 taxa, we identified three significantly increased taxa in ASD patients: Eubacteriales, *Klebsiella*, and *Clostridium*. Additionally, there were enriched trends of *Oscillospira*, *Dorea*, and *Collinsella*, and depleted trends of *Streptococcus*, *Akkermansia*, *Coprococcus*, and *Dialister*. These findings suggest that the disrupted intestinal microecology and functional changes in ASD are characterized by an enrichment of pro-inflammatory genera, a reduction of specific probiotics, lactic acid-producing and utilizing bacteria, and an imbalance of anti-inflammatory butyrate-producing bacteria. Substantial heterogeneity across studies concerning demographics and methodologies was also observed.

**Conclusion:**

This systematic review contribute to a further understanding of the role of gut microbiota in ASD and support the development of microbiota-based diagnostic and therapeutic strategies for ASD.

## 1 Introduction

Autism spectrum disorder (ASD) is a complex neurobiological disorder characterized by altered social interaction and repetitive and stereotyped behavior (Lord et al., 2020). In recent decades, the prevalence of ASD has significantly increased worldwide. Recent epidemiological studies have reported that there is one morbidity in every 139 children (Olusanya et al., 2023). Due to the persistence of ASD from childhood to adulthood, the disease imposes a substantial and increasing burden on public health and socio-economy (Buescher et al., 2014; Li et al., 2022). Notably, 78% of individuals with ASD report gastrointestinal symptoms, with the most common symptoms including constipation and abdominal pain. Furthermore, gastrointestinal issues may exacerbate behavioral problems in children with ASD, such as difficulties in social interaction and self-injurious behaviors (Deng et al., 2022). Currently, the pathogenesis of ASD remains unclear, and its clinical diagnosis mainly relies on subjective identification of the symptoms; effective medical interventions are also limited. Therefore, it is of crucial clinical value to identify biomarkers sensitive to the pathological processes of ASD and to develop a novel therapy.

The gut microbiota is a microbial community closely associated with various physiological processes in the human body. Gut microbiota dysbiosis has been widely implicated in the pathogenesis of neurological disorders through the “microbiota-gut-brain” axis (Strati et al., 2017), which has been recognized to regulate the functions of the gastrointestinal and central nervous systems through a bidirectional communication system between the gut and the brain. This involvement may play a potential role in the pathogenesis of depression, Alzheimer’s disease, and Parkinson’s disease through mechanisms such as immune activation and the action of microbial metabolites, influencing emotions and contributing to the development of these disorders (Margolis et al., 2021). Previous studies have indicated that the pathways by which the gut microbiota influences social behavior and brain physiology include immune activation (Buffington et al., 2021; Furness et al., 1999), production of microbial peptides, metabolites, and multiple neuroregulators and neurotransmitters (Sherwin et al., 2019). Further study has demonstrated that fecal microbiota transplantation can restore gut microbial balance in ASD patients, alleviate gastrointestinal symptoms, and potentially improve core autism symptoms (Warner, 2019). Thus, the crucial role of the gut-brain axis in the pathophysiology of neurological disorders appears to be driven by the ecology and function of the gut microbiota (Collins et al., 2012). Additionally, gastrointestinal issues in ASD children were reported to be more frequent and severe compared to neurotypical children (Vuong and Hsiao, 2017). Thus, it is suggested that ASD-related symptoms may be associated with gastrointestinal dysfunction, possibly resulting from the disruption of the microbiota-gut-brain axis (Vuong and Hsiao, 2017). Therefore, identifying key microbial taxa can aid in understanding ASD etiology and identifying biomarkers for clinical use, as well as identifying new therapeutic targets.

In recent years, with the rapid development of high-throughput technologies, such as 16S rRNA sequencing and metagenomic sequencing, increasing studies have investigated gut microbiota alterations in ASD patients. Previous studies have demonstrated specific changes in the gut microbiota of individuals with ASD (Strati et al., 2017). However, results from currently available data showed poor consistency due to the high heterogeneity of the patients included (such as varying disease severity, gastrointestinal symptoms, residence, etc.). Further investigation is necessary to explore the relationship between the gut microbiota and ASD and to identify the microbial markers across different studies.

In this study, we aim to comprehensively assess the changes in gut microbial diversity and taxa in ASD patients and to identify consistently altered gut microbiota in ASD. We performed a most up-to-date analysis of clinical studies on the gut microbiota perturbations in ASD patients using the vote counting statistical method. Also, we analyzed the potential impacts of confounding factors on the gut microbiota. These findings contribute to further understanding the role of gut microbiota in ASD and developing new microbiota-based diagnosis and therapy for ASD patients.

## 2 Materials and methods

### 2.1 Search strategy

This study was conducted following the Preferred Reporting Items for Systematic Reviews and Meta-analyses (PRISMA) guidelines (Page et al., 2021). In accordance with the PICO criteria, the target population comprised individuals clinically diagnosed with ASD, regardless of ongoing medication use (e.g., antipsychotics or gastrointestinal drugs); interventions were defined as any microbiota-related treatments (e.g., probiotics, antibiotics) or absence of such targeted interventions; the comparisons were neurotypical controls or placebo; outcomes were symptoms, gut microbiota composition, 16S rRNA, or metagenome sequencing results.

Electronic literature searches were conducted in the PubMed, Web of Science, Cochrane Library and Embase databases up to February 16th, 2023 to retrieve relevant literature on the gut microbiota composition in ASD patients. The details of the search strategy can be found in Supplementary Table 1.

### 2.2 Data inclusion and exclusion

The eligibility of all studies was independently screened and evaluated by two researchers (Tao X and Li Z), with intervention from a third reviewer (Wang D) in case of any disagreements.

The studies eligible for data extraction were required to satisfy the following criteria: (1) original human studies; (2) investigating gut bacteria in children diagnosed with autism or ASD; (3) gut microbiota were determined by high-throughput sequencing, including 16S rRNA or metagenomic sequencing; (4) reported the differences in microbial diversity indices (α and β diversity) or significantly different gut microbiota between ASD patients and healthy control group.

The exclusion criteria included animal experiments, studies without available control groups, secondary analyses (such as meta-analysis), literature reviews and conference abstracts. The detailed exclusion records are provided in Supplementary Table 2.

### 2.3 Study quality assessment

To assess study quality, the Newcastle-Ottawa scale (NOS) was used, and the assessment was performed by Tao X and Li Z. The NOS assigns a maximum of 9 points based on three quality parameters, including selection, comparability and outcome (Wells et al., 2015). According to the NOS grading in previous reviews, we classified studies as high (< 5 stars), moderate (5–7 stars) and low risk of bias (8–9 stars) (Pizzol et al., 2021).

### 2.4 Data extraction

Data extraction was performed by Tao X using a pre-determined form, which underwent independent validation by another two researchers (Li Z and Wang D). The extracted data include basic information about the studies [such as study title, diagnostic criteria, disease severity, intervention, sample type, sample size, age, sex, Body Mass Index (BMI), sequencing methods and amplicon region] and information on the differential gut microbiota reported by the studies (microbiota name, classification level, up/down regulated changes, NCBI taxonomy ID, lineage, comparison groups, diversity assessment indices and their alterations).

### 2.5 Subgroup analyses

To investigate the potential effects of the confounding factors on the gut microbiota, we further stratified the differential microbiota data based on disease severity, functional gastrointestinal symptoms, countries or regions and sequencing methods. Subsequent statistical analyses were performed within each subgroup category and compared between subgroups within the same category or between a subgroup and the entire population.

### 2.6 Data analysis

Although the optimal method for integrating the differential microbiota data was combining the average values, *P*-values, or raw data of each study, it is challenging to conduct a meta-analysis due to the lack of these values in most of the included studies. Therefore, we performed a vote counting method to analyze whether microbiota were consistently up- or down-regulated across studies. The vote counting method is a statistical approach that identifies consistently altered microbial taxa by voting for their upregulation or downregulation across multiple studies. Specifically, healthy controls served as the baseline, and each microbial taxon was assigned a vote (+1 or −1) based on its reported regulation in ASD patients relative to healthy controls. The sum of these votes was used to determine the consistency of alteration. This method facilitated the identification of candidate biomarkers that are likely to be validated by independent tests (Rikke et al., 2015).

Considering that the reporting frequency of microbiota can be potentially diluted, we conducted the vote counting analysis for differential microbiota reported in three or more studies. During this process, each microbial taxon was assigned a weight of “+1” or “−1” when reported as significantly up-regulated or down-regulated, respectively. Then, we calculated the vote counting statistic (VCS) for each taxon by summing the individual scores. Higher or lower VCS values indicated that more studies reported significant up-regulation or down-regulation of the microbiota in ASD patients compared to HC.

We then used a binomial distribution to assess whether the change of each microbial taxon was statistically significant, assuming a probability of 50% of up-regulation or down-regulation for each microbe in each study (Goveia et al., 2016). The binomial tests were performed using the binom.test function in R (v4.0.4).^1^ We calculated one-sided *P*-values for gut microbiota reported in three or more datasets. A *P*-value less than 0.05 was considered to be statistically significant.

## 3 Results

### 3.1 Characteristics of the included studies

**FIGURE 1:**
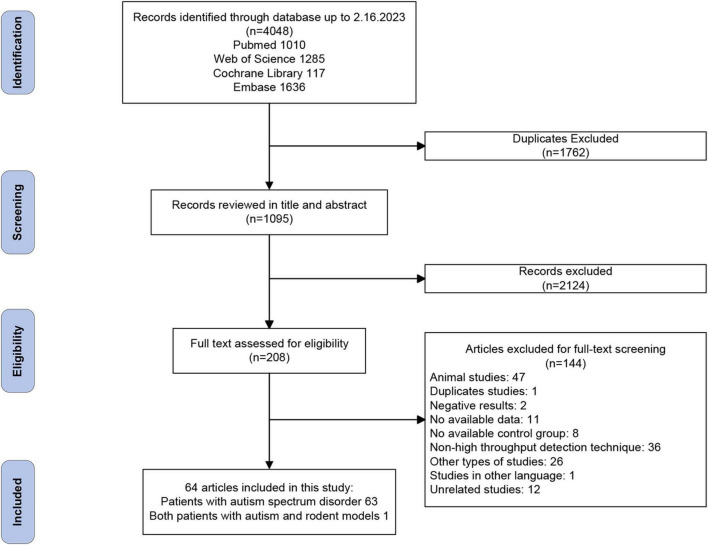
PRISMA flowchart.

The flowchart for data screening is illustrated in Figure 1. Among the 4,048 records retrieved from the databases, 2,322 remained after removal of duplicates. Based on our eligibility criteria, 208 articles were selected. Of these, 144 articles were excluded after a full-text screening, resulting in the inclusion of 64 articles. A quality evaluation was performed using NOS on the 64 articles, as shown in Supplementary Table 3. The result showed that 33 studies (51.6%) have a low risk of bias, and the other 31 (48.4%) were classified as having a moderate risk of bias.

Of the 64 included articles, 26 (40.6%) were conducted in China, 17 in the United States of America (USA) (26.6%), and the remaining 21 (32.8%) in other countries, including the United Kingdom, Spain, Slovakia, Russia, etc. The 64 case-control studies generated 80 comparisons, involving 3,359 patients and 2,632 controls. The sample size of each study ranged from 6 to 143. ASD cases were mainly diagnosed using The Diagnostic and Statistical Manual of Mental Disorders, fourth/fifth edition (DSM-IV/V) or International Classification of Diseases (ICD-10), with 21 (32.8%) studies using the Childhood Autism Rating Scale (CARS) to assess the symptoms of patients with ASD. Regarding the sequencing methods, 16S rRNA amplicon sequencing was the most common method (50/64, 78.1%), followed by metagenomic sequencing (9/64, 14.1%), and 3 studies (4.7%) used both the above two methods. The detailed information for each study is provided in Supplementary Table 4.

### 3.2 Alterations of alpha and beta diversity in ASD

**FIGURE 2:**
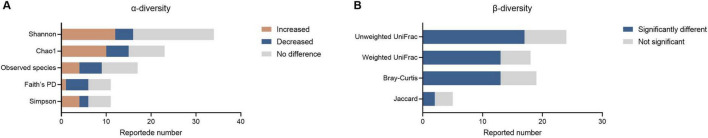
Differences in α and β-diversity in patients with ASD compared to controls. **(A)** Differences in α-diversity between patients with ASD and controls. **(B)** Differences in β-diversity between patients with ASD and controls.

For α-diversity analysis, 45 studies reported changes in microbial α-diversity, resulting in 115 α-diversity analyses (Supplementary Table 5). Shannon index (34/115, 29.6%), Chao1 (23/115, 20.0%), Observed species (17/115, 14.8%), Faith’s PD (11/115, 9.6%) and Simpson (11/115, 9.6%) were the most frequently reported indices (Figure 2A). Almost half of the α-diversity analyses (54/115, 47.0%) found no significant difference between patients with ASD and controls, while only 31.3% (36/115) and 21.7% (25/115) of analyses indicated increased or decreased α-diversity in ASD, respectively. Therefore, based on our synthesized data, we did not find strong evidence for a difference in the microbial α-diversity between autistic patients and healthy controls.

For β-diversity analysis, 38 studies reported differences in β-diversity between autistic patients and healthy individuals, resulting in 66 β-diversity analysis outcomes (Supplementary Table 5). The employed analysis methods included Bray–Curtis (19/66, 28.8%), weighted (18/66, 27.2%) and unweighted UniFrac (24/66, 36.3%), along with Jaccard similarity index (5/64, 7.6%) (Figure 2B). In total, more than half of β-diversity analyses (45/66, 68.2%) found significantly different results, while only 25.76% of analyses indicated no significant differences (21/64). These data suggest a changing tendency of the gut microbiota composition in autistic patients compared to neurotypical controls.

### 3.3 Differentially abundant microbial taxa in ASD

**FIGURE 3:**
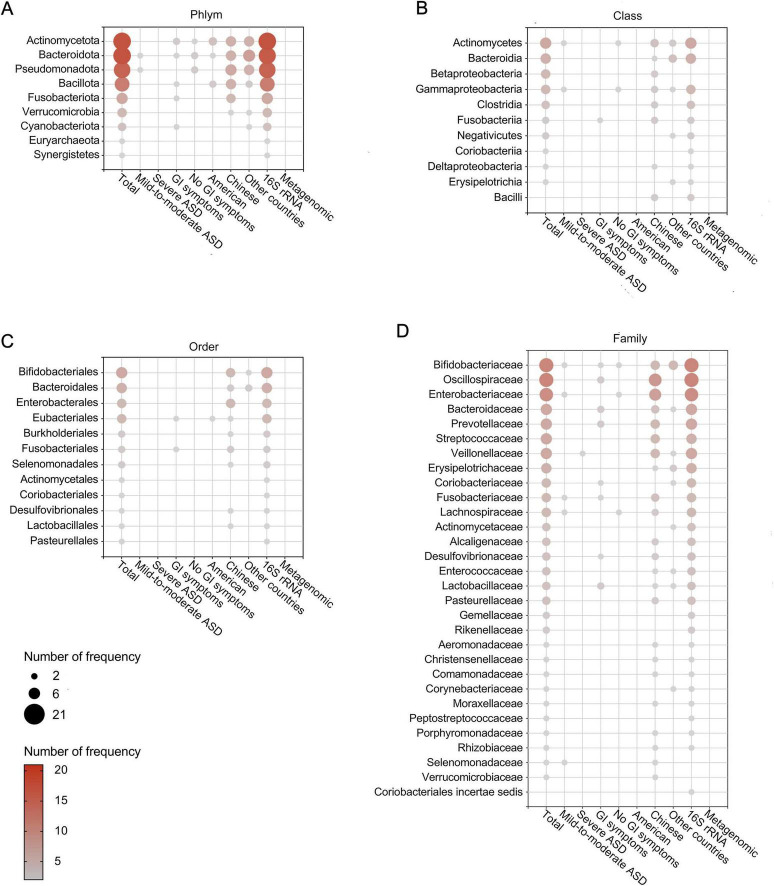
Differentially abundant taxa at the phylum **(A)**, class **(B)**, order **(C)**, and family **(D)** levels in patients with ASD. Only the abundant taxa that were reported by ≥ 2 studies are shown. Each circle represents a taxon, and the size of each circle represents the number of studies that reported the taxa.

**FIGURE 4:**
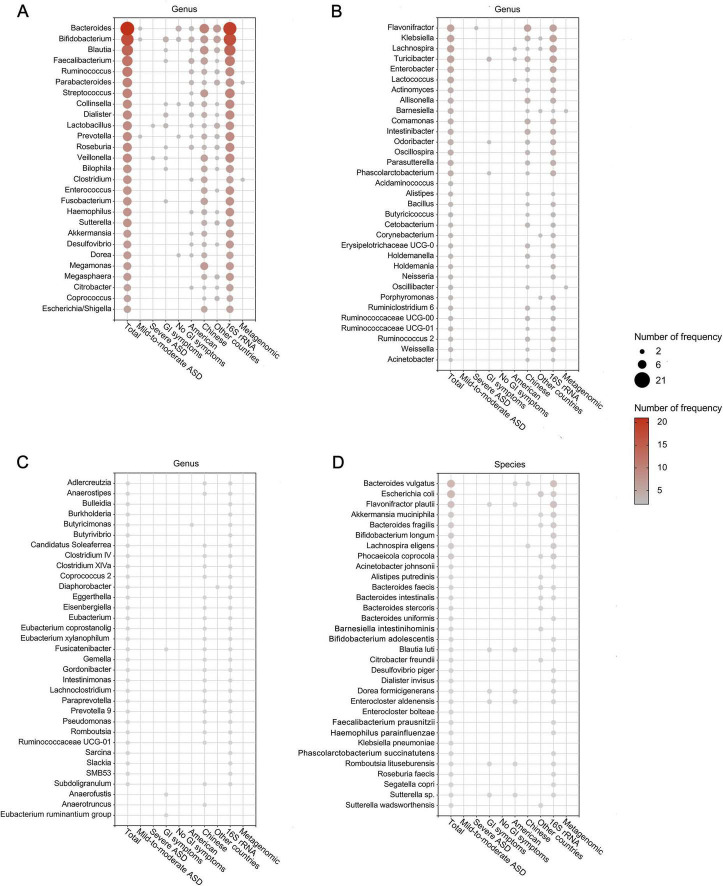
Differentially abundant taxa at the genus **(A–C)** and species **(D)** levels in patients with ASD. Only the abundant taxa that were reported by ≥ 2 studies are shown. Each circle represents a taxon, and the size of each circle represents the number of studies that reported the taxa.

After removing duplicates, we summarized microbial findings reported by ≥ 2 studies to avoid the risk of false positives. Overall, 189 microbial taxa were found to be differentially abundant in patients with ASD, spanning 10 phyla, 11 classes, 12 orders, 30 families, 93 genera, 31 species, 1 no rank taxon and 1 microbe ratio (Figures 3, 4 and Supplementary Table 6). As Figures 3, 4 showed, phyla Actinomycetota and Bacteroidota, class Actinomycetes, order Bifidobacteriales, families Bifidobacteriaceae and Oscillospiraceae, genera *Bacteroides*, *Bifidobacterium*, *Blautia* and *Faecalibacterium* and species *Bacteroides vulgatus* and *Escherichia coli* were the most frequently reported taxa. Supplementary Figure 1 displayed the lineage of 109 taxa, which were reported in ≥ 3 datasets, illustrating that the differential genera were mainly assigned to Oscillospiraceae at the family level and mainly derived from Bacillota at the phylum level.

**FIGURE 5:**
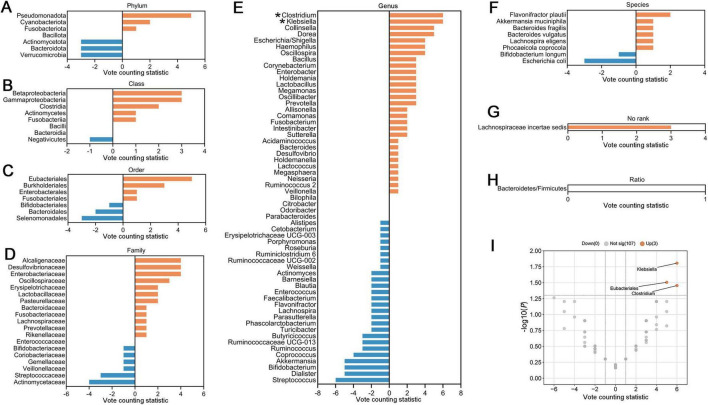
Vote counting statistics for differential microbial taxa that were reported by ≥ 3 studies at phylum **(A)**, class **(B)**, order **(C)**, family **(D)**, genus **(E)**, species **(F)**, no rank **(G)**, and ratio **(H)** levels. An asterisk (*) represents the difference was statistically significant. The vote counting statistic for each taxon is represented by orange and blue bars. **(I)** Volcano plots of abundant taxa resulting from vote counting analyses. The *x*-axis indicates the vote counting statistic, and the *y*-axis shows the –log10 (*P*-value). The plot contains 107 gray dots (non-significant) and 3 red points (significantly up-regulated). Some gray points overlap due to identical vote counting statistic and *P*-values.

To further identify gut microbial biomarkers with consistent alterations for ASD compared with healthy controls, we conducted the vote counting analysis on the microbial taxa reported in ≥ 3 datasets (Figures 5A–H). We identified that only three taxa exhibited consistent changes in ASD patients across the studies. Specifically, order Eubacteriales (VCS = 5, *P* = 0.031), and genera *Klebsiella* (VCS = 6, *P* = 0.016) and *Clostridium* (VCS = 6, *P* = 0.035) were significantly enriched in autistic patients (Figure 5I). The enrichment trends were observed in families Alcaligenaceae and Desulfovibrionaceae, and genera *Dorea*, *Oscillospira* and *Collinsella* in ASD (*P* < 0.1), while Actinomycetaceae at the family level, *Streptococcus*, *Coprococcus*, *Akkermansia* and *Dialister* at the genus level, tended to be depleted in patients with ASD (*P* < 0.1).

### 3.4 Effects of potential confounders on microbial alterations

**FIGURE 6:**
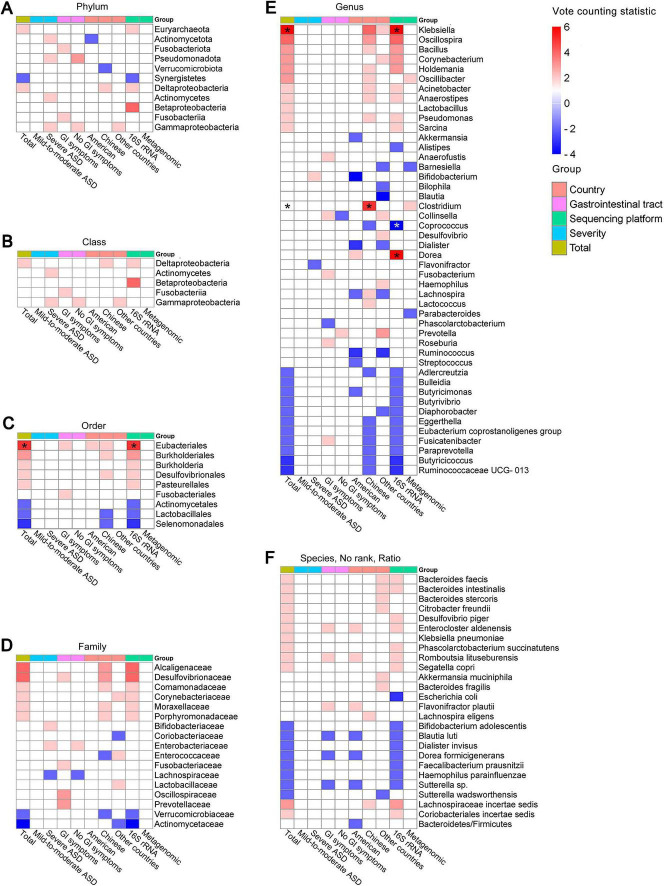
Heatmap of abundant taxa at phylum **(A)**, class **(B)**, order **(C)**, family **(D)**, genus **(E)**, and species, no rank and ratio **(F)** levels in the entire patient group and different subgroups of patients with ASD. Only taxa that were concordantly reported by ≥ 2 studies were presented here. Red represents that the taxon was consistently enriched, while blue represents that the taxon was consistently depleted across studies. An asterisk (*) represents the difference was statistically significant.

To explore the effects of potential confounding factors on microbial alterations, we further performed subgroup analyses. We categorized the included studies based on the disease severity, the presence or absence of functional gastrointestinal disorders, the study region and sequencing methods. We summarized the alterations of gut microbiota, which were consistently altered as reported by ≥ 2 studies in the subgroups (Figure 6), and primarily analyzed the differential taxa at the genus level.

#### 3.4.1 Severity of ASD

To investigate the effects of the severity of ASD on gut microbiota, we stratified the studies based on the CARS score of patients to investigate the influence of disease severity. Patients with a mean CARS score ranging from 30 to 35 were classified as the mild-to-moderate group (4 studies), and those with a mean score of 36 or higher were classified as the severe group (4 studies) (Schopler et al., 1980). In the mild-to-moderate patients, we observed consistently decreased Flavonifractor and consistently increased *Bifidobacterium* in two reports. For the severe ASD group, *Lactobacillus* was found to be consistently increased in two reports. However, owing to the limited studies reporting CARS scores, these observations should be considered preliminary, and more research is needed to explore the differences in gut microbiota for ASD patients with different severity.

#### 3.4.2 Functional gastrointestinal symptoms

Gastrointestinal disorders rank as one of the prevalent medical comorbidities observed in individuals with ASD. We analyzed the microbiota results in ASD patients with (10 studies) and without gastrointestinal disorders (4 studies), respectively. For ASD patients with gastrointestinal disorders, we identified that *Fusobacterium* and *Roseburia* were up-regulated consistently (Supplementary Figure 2A). Depleted *Collinsella* and Prevotella were observed in patients without gastrointestinal diseases. Notably, *Collinsella* abundance was significantly elevated in ASD patients with gastrointestinal disorders compared to those without such symptoms (Wong et al., 2022). This genus, known for its ability to convert primary bile acids into pro-inflammatory secondary bile acids and compete with butyrate-producing bacteria (Guzior and Quinn, 2021; Long et al., 2023), may serve as both a biomarker for gastrointestinal comorbidity and a potential contributor to ASD pathophysiology.

#### 3.4.3 Study region

We explored the impact of the study region on microbial alterations. Due to the imbalanced availability of studies by region (with most studies conducted in the USA and China), we compared the gut microbiota between autistic patients in China and those in the United States. The vote counting results showed that in studies from China, *Clostridium* was significantly up-regulated (VCS = 5, *P* = 0.031) (Supplementary Figure 2B). In addition, clustering according to the region identified several taxa that were altered only in studies from China: consistent enrichment of Acinetobacter, Anaerostipes, Lachnospira, *Lactococcus* and Pseudomonas studies, and depletion of *Adlercreutzia*, Eggerthella, Fusicatenibacter and Paraprevotella reported in 2 of 25 studies (Supplementary Figure 2B). In studies from the USA, extra alterations were observed for *Dialister*, Ruminococcus and *Bifidobacterium* reported in 3 of 17 studies, as well as Butyricimonas and Lachnospira in 2 studies (Supplementary Figure 2C). These differences driven by factors such as dietary patterns and genetics highlighted the need to distinguish the gut microbiome among different regions as more evidence becomes available.

#### 3.4.4 Sequencing methods

Further, we explored the effect of the sequencing method and summarized the microbial changes resulting from 52 studies using 16S rRNA amplicon sequencing and 9 studies using metagenomic sequencing. The vote counting analyses suggested that in addition to consistent increases of *Klebsiella* (VCS = 6, *P* = 0.016) and Eubacteriales (VCS = 5, *P* = 0.031) which also exhibited differences in the overall results, we also found a significant up-regulation of *Dorea* (VCS = 6, *P* = 0.016), and a reduction of *Coprococcus* (VCS = −5, *P* = 0.031) only obtained from 16S rRNA sequencing (Supplementary Figures 3, 4). Although we found no statistically differential microbiota from studies using metagenomic sequencing, 2 studies reported consistent up-regulation in *Clostridium* and *Oscillibacter* and down-regulation in *Barnesiella* and *Parabacteroides*.

## 4 Discussion

To our knowledge, this is the most comprehensive investigation, including the most up-to-date reports, including 64 studies, and found 189 differential gut microbial taxa that were reported by more than 2 studies. We used vote counting analysis to evaluate the reproducibility and stability of potential gut microbial biomarkers.

There is an assumption that a higher diversity of gut microbiota symbolizes a healthy state (Shade, 2017), while a lower α-diversity is considered a marker of disease status (Chang et al., 2008). Our integrative analysis found no strong evidence supporting alterations in α-diversity for ASD patients. However, β-diversity was observed to be distinct in individuals with ASD compared to healthy controls, which is consistent with previous studies.

**FIGURE 7:**
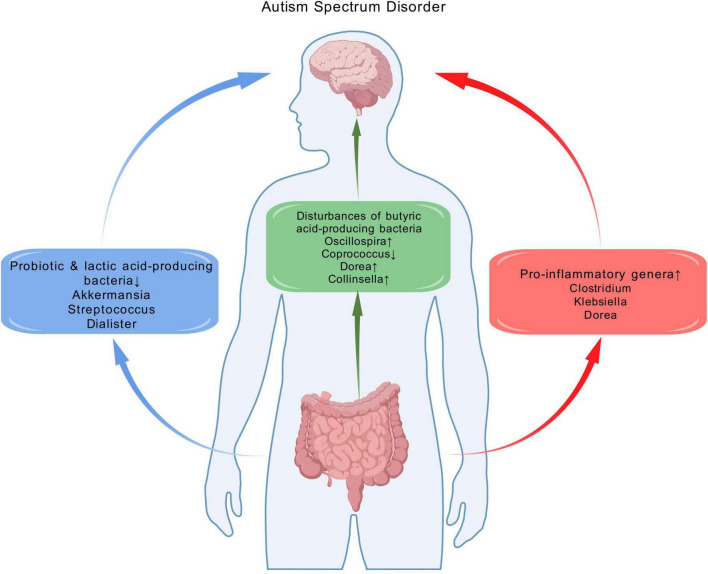
The alterations of gut microbiota and potential functional implications of bacterial genera implicated as different in ASD in this systematic review. These alterations are potentially related to the pathophysiology of ASD, as they involve the enrichment of pro-inflammatory genera, reduction of specific probiotics and lactic acid–producing bacteria, as well as the disturbance of butyrate-producing bacteria. Created with biogdp.com.

To identify effector microbial biomarkers, we explored the relevant functions of the genus-level taxa to elucidate potential commonalities in the differential taxa associated with ASD (Figure 7). Inflammation mediated by microbiota composition is a key element in ASD (Iglesias-Vázquez et al., 2020). In agreement with previous findings, our study indicated that pro-inflammatory genera, including *Clostridium*, *Klebsiella* and *Dorea*, were enriched. Among these, *Clostridium* and *Klebsiella* metabolize amino acids and proteins through putrefaction (Kaur et al., 2017), increasing higher concentration of putrefaction products, including ammonia, sulfide and biogenic amines, which are implicated in intestinal inflammation (Cheung et al., 2019). *Dorea* has a wide range of metabolic functions, including the degradation of mucin, which is essential for maintaining the normal state of the gut mucosal layer (Abujamel et al., 2022). Lower abundance of mucin in the gut results in thinning of the mucosal layer, increasing gut permeability (Zhang et al., 2019) and inflammatory responses (Maes et al., 2008; Wang et al., 2011).

Another noteworthy observation is the down-regulation of the probiotic *Akkermansia*, a mucosin-producing microbiome (Agarwala et al., 2021). Meanwhile, the down-regulation of lactic acid-producing bacteria *Streptococcus* and *Dialister* were also observed. Lactic acid contributes to suppressing the growth of pathogens such as *E. coli* across the epithelium, reducing inflammation in the gut (Xu et al., 2019). The decrease of lactic acid-producing bacteria may cause lactic acid dysregulation, resulting in intestinal inflammation. These findings confirmed the role of decreased probiotics and lactic acid-producing bacteria in ASD.

Butyric acid serves as an energy source for intestinal epithelial cells. It inhibits the release of pro-inflammatory cytokines and plays a crucial role in gut immune homeostasis (Hoffman, 1989; Lewis et al., 2010). Moreover, previous studies have described its ability to improve repetitive behavior in BTBR mice (Kratsman et al., 2016), suggesting its positive benefits for ASD. Therefore, the disturbances of butyric acid-producing bacteria may contribute to intestinal inflammation (Iglesias-Vázquez et al., 2020). Our study indicated disturbances of butyric acid-producing bacteria, including up-regulated *Oscillospira* and down-regulated *Coprococcus*. Meanwhile, we observed an increase in *Dorea* and *Collinsella*, which inhibit the absorption of short chain fatty acids (including butyric acid) in the liver. This has further impacts on brain function and behavior (Ding et al., 2020).

Among the confounders that led to inconsistencies in microbial composition across studies, we categorized the included studies with available data into subgroups based on ASD severity, presence of gastrointestinal symptoms, study region and sequencing method. Despite previous findings that *Lactobacillus* and *Bifidobacterium* can prevent intestinal inflammation and improve ASD symptoms (Ehrlich et al., 2020; Proença et al., 2018; Xu et al., 2019), our results showed that *Lactobacillus* was consistently up-regulated in severe ASD patients, while *Bifidobacterium* was consistently up-regulated in mild to moderate patients. However, due to the limited number of each subgroup, we were not able to analyze the association between ASD severity and gut microbiota. In ASD with gastrointestinal symptoms, consistent up-regulation of *Fusobacterium* and *Roseburia* was observed. *Fusobacterium Lipopolysaccharides* induce inflammatory conditions through Toll-like receptors and NFκB pathways (Shaaban et al., 2018; Bashir et al., 2016). Enrichment of *Roseburia* is related to the high concentration of glutamate, a neurotoxin that may cause neuropsychiatric disorders pathophysiology, including ASD (Won et al., 2012; Lukjancenko et al., 2012). We suggest that there may be a potential link between gastrointestinal symptoms and gut microbiota dysbiosis in people with ASD. Therefore, further studies are needed to clarify this relationship. In the geographical subgroup, we observed significant heterogeneity in the alteration of gut microbiota, especially in the Chinese group. For instance, *Lactococcus*, which enhances immune response (Yang and Chang, 2014), consistently up-regulated, while *Adlercreutzia* which may induce ASD by interfering with microglial cell function through equol production (Laue et al., 2020), consistently down-regulated. Although several studies have highlighted the influence of genetics and diet on the composition and function of the gut microbiota (Cho and Blaser, 2012; David et al., 2014; Derrien et al., 2019), in our current systematic review, we identified that data regarding the interaction between genetic forms of ASD and gut microbiota changes were limited. Consequently, the potential impact of specific genetic and dietary confounders on gut microbiota could not be adequately assessed. Furthermore, given the substantial heterogeneity across studies concerning patient demographics, it was challenging to isolate the distinct contributions of genetic factors from other potential confounders. Therefore, it is necessary to develop independent microbiome databases stratified by geographical regions and genetic profiles.

## 5 Limitations

Our study has certain limitations. Firstly, the gut microbiota begins to resemble at around three years, but the evidence proves that there is a further maturation in later childhood (Yatsunenko et al., 2012). Since our study included individuals across a wide range of ages (from 2 to 52 years old), differences in gut microbiome in patients with different ages may impact the overall results. Secondly, while the application of vote counting analysis can effectively analyze large-scale data, it cannot identify new gut microbiota. However, considering that only a few of the included studies provided raw data, this method remains the best approach for conducting such a semi-quantitative analysis. The lack of raw data also restricts our ability to perform more advanced statistical analyses and explore potential interactions between different microbial taxa. Thirdly, this study only focused on the bacterial component of the gut microbiota and did not investigate the mycobiota. Although evidence suggests that the mycobiota may also play a role in ASD and other disorders, due to resource and data constraints, we were unable to explore this aspect. Additionally, medications are significant covariates of the gut microbiome (Vujkovic-Cvijin et al., 2020). However, since most included studies didn’t clarify the details of medications for ASD patients (Feroe et al., 2021), it is not available to eliminate the effects of medications on gut microbiota in our study. Due to the limited availability of data, other potential confounders, such as gender and sequencing samples, were not investigated. With more studies providing larger amounts of data, these potential confounders should be explored in the future.

## 6 Conclusion

In conclusion, our study indicated that there was no substantial evidence to support significant differences in α-diversity between ASD patients and healthy controls. In contrast, β-diversity tends to be distinct in ASD patients. Among 189 differential differentially abundant taxa, we identified three significantly altered taxa in ASD patients, as well as 10 genera with changing trends. The findings suggested that gut microbiota changes in ASD patients were characterized by an enrichment of pro-inflammatory genera, reductions of specific probiotics, and lactic acid-producing bacteria, as well as an imbalance of anti-inflammatory butyrate-producing bacteria. Furthermore, the heterogeneity of ASD patients had a significant impact on the measurement results of gut microbiota. This study contributes to identifying microbial biomarkers of ASD and developing microbiota-based diagnosis and therapy for ASD.

## Data Availability

The raw data supporting the conclusions of this article will be made available by the authors, without undue reservation.

## Footnotes

1
https://www.rproject.org/
