# Supplementary material for: Perturbations in gut microbiota in autism spectrum disorder: a systematic review
Source: Front Neurosci. 2025 May 16;19:1448478. doi: 10.3389/fnins.2025.1448478 (PMC12122542; doi:10.3389/fnins.2025.1448478)

***Supplementary material***

**Perturbations in gut microbiota in autism spectrum disorder: a systematic review**

**Xiangkun** **Tao^1†^, Zhuocan Li^1†^, Dongfang Wang^1,2,3^, Juncai Pu^1,2,4^, Yiyun Liu^1,2,3^, Siwen Gui^1,2,3^, Xiaogang Zhong^1,2,5^, Dan Yang^1^, Haipeng Zhou^1^, Wei Tao^1^, Weiyi Chen^1,4^, Xiaopeng Chen^1,4^, Yue Chen^1,4^, Xiang Chen^1,4^, Peng Xie^1,2,3,4^***

^1^NHC Key Laboratory of Diagnosis and Treatment on Brain Functional Diseases, The First Affiliated Hospital of Chongqing Medical University, Chongqing, 400016, China

^2^Jinfeng Laboratory, Chongqing, 401329, China

^3^Chongqing Institute for Brain and Intelligence, Chongqing, 401336, China

^4^Department of Neurology, The First Affiliated Hospital of Chongqing Medical University, Chongqing, 400016, China

^5^College of Basic Medicine, Chongqing Medical University, Chongqing, 400042, China

† These authors contributed equally to this work

***Correspondence:**

Peng Xie:

xiepeng@cqmu.edu.cn

**Table S1.** Search Strategy of each database.

| **Key terms for search of PubMed** | |
| --- | --- |
| #1 | gut microbiota[MeSH Terms] OR ((gut[Title/Abstract] OR gastrointestin*[Title/Abstract] OR intestin*[Title/Abstract] OR fecal[Title/Abstract] OR faecal[Title/Abstract] OR feces[Title/Abstract] OR stool[Title/Abstract]) AND (microbiota[Title/Abstract] OR microbiome[Title/Abstract] OR microflora[Title/Abstract] OR flora[Title/Abstract] OR bacteria[Title/Abstract] OR microbi*[Title/Abstract] OR microbe*[Title/Abstract])) OR probiotic*[Title/Abstract] OR prebiotic*[Title/Abstract] OR synbiotic*[Title/Abstract] |
| #2 | 16S rRNA[MeSH Terms] OR metagenomics[MeSH Terms] OR metagenome[MeSH Terms] OR 16S rRNA[Title/Abstract] OR 16S rDNA[Title/Abstract] OR metagenomic*[Title/Abstract] OR metagenome*[Title/Abstract] OR metaproteomic*[Title/Abstract] |
| #3 | #1 OR #2 |
| #4 | autism spectrum disorder[MeSH Terms] OR pervasive development disorder[MeSH Terms] OR autism[Title/Abstract] OR autistic[Title/Abstract] OR asperger*[Title/Abstract] OR pervasive development*[Title/Abstract] |
| #5 | #3 AND #4 |
| **Key terms for search of Embase** | |
| #1 | ‘intestine flora’/exp OR ((gut OR gastrointestin* OR intestin* OR fecal OR faecal OR feces OR stool):ti,ab AND (microbiota OR microbiome OR microflora OR flora OR bacteria OR microbi* OR microbe*):ti,ab) OR (probiotic* OR prebiotic* OR synbiotic*):ti,ab |
| #2 | ‘RNA 16S’/exp OR ‘DNA 16S’/exp OR ‘metagenomics’/exp OR ‘metagenome’/exp OR ‘metaproteomics’/exp OR (‘16S rRNA’ OR ‘16S rDNA’ OR metagenomic* OR metagenome* OR metaproteomic*):ti,ab |
| #3 | #1 OR #2 |
| #4 | ‘autism’/exp OR (autism OR autistic OR asperger* OR ‘pervasive development*’):ti,ab |
| #5 | #3 AND #4 |
| **Key terms for search of Web of Science** | |
| #1 | (TS=(gut OR gastrointestin* OR intestin* OR fecal OR faecal OR feces OR stool)) AND TS=(microbiota OR microbiome OR microflora OR flora OR bacteria OR microbi* OR microbe*) |
| #2 | TS=(probiotic* OR prebiotic* OR synbiotic*) |
| #3 | TS=(“16S rRNA” OR “16S rDNA” OR metagenomic* OR metagenome* OR metaproteomic*) |
| #4 | #1 OR #2 OR #3 |
| #5 | TS=(autism OR autistic OR asperger* OR “pervasive development*”) |
| #6 | #4 AND #5 |
| **Key terms for search of Cochrane Library** | |
| #1 | MeSH descriptor: [gastrointestinal microbiome] explode all trees |
| #2 | (gut OR gastrointestin* OR intestin* OR fecal OR faecal OR feces OR stool):ti,ab,kw AND (microbiota OR microbiome OR microflora OR flora OR bacteria OR microbi* OR microbe*):ti,ab,kw |
| #3 | (probiotic* OR prebiotic* OR synbiotic*):ti,ab,kw |
| #4 | #1 OR #2 OR #3 |
| #5 | MeSH descriptor: [RNA, ribosomal, 16S] explode all trees |
| #6 | MeSH descriptor: [metagenomics] explode all trees |
| #7 | MeSH descriptor: [metagenome] explode all trees |
| #8 | ‘16S rRNA’ OR ‘16S rDNA’ OR metagenomic* OR metagenome* OR metaproteomic* |
| #9 | #5 OR #6 OR #7 OR #8 |
| #10 | #4 OR #9 |
| #11 | MeSH descriptor: [autism spectrum disorder] explode all trees |
| #12 | MeSH descriptor: [child development disorders, pervasive] explode all trees |
| #13 | (autism OR autistic OR asperger* OR pervasive development*):ti,ab,kw |
| #14 | #11 OR #12 OR #13 |
| #15 | #10 AND #14 |

**Table S2.** Exclusion reasons for full‐text reports.

| **Study Name** | **Title** | **Excluded reasons** |
| --- | --- | --- |
| Abuaish S 2021 | Fecal Transplant and Bifidobacterium Treatments Modulate Gut Clostridium Bacteria and Rescue Social Impairment and Hippocampal BDNF Expression in a Rodent Model of Autism | Non-high throughput detection technique |
| Abuaish S 2022 | The Efficacy of Fecal Transplantation and Bifidobacterium Supplementation in Ameliorating Propionic Acid‑Induced Behavioral and Biochemical Autistic Features in Juvenile Male Rats | Non-high throughput detection technique |
| Abujamel TS 2022 | Different Alterations in Gut Microbiota between Bifidobacterium longum and Fecal Microbiota T ransplantation T reatments in Propionic Acid Rat Model of Autism | Animal studies |
| Adams JB 2011 | Gastrointestinal flora and gastrointestinal status in children with autism–comparisons to typical children and correlation with autism severity | Non-high throughput detection technique |
| Afroz KF 2021 | Altered gut microbiome and autism like behavior are associated with parental high salt diet in male mice | Animal studies |
| Alfawaz HA 2022 | Protective Effects of Bee Pollen on Multiple Propionic Acid-Induced Biochemical Autistic Features in a Rat Model | Non-high throughput detection technique |
| Alghamdi MA 2022 | Bee Pollen and Probiotics May Alter Brain Neuropeptide Levels in a Rodent Model of Autism Spectrum Disorders | Other types of studies |
| Alhusaini A 2022 | Acetyl-L-carnitine and/or liposomal co-enzyme Q10 prevent propionic acid-induced neurotoxicity by modulating oxidative tissue injury; inflammation; and ALDH1A1-RA-RARα signaling in rats | Non-high throughput detection technique |
| Alonazi M 2022 | Psychobiotics improve propionic acid-induced neuroinflammation in juvenile rats; rodent model of autism | Non-high throughput detection technique |
| Alookaran J 2022 | Fungi: Friend or Foe? A Mycobiome Evaluation in Children with Autism and Gastrointestinal Symptoms | Unrelated studies |
| Alsubaiei SRM 2022 | Independent and Combined Effects of Probiotics and Prebiotics as Supplements or Food-Rich Diets on a Propionic-Acid-Induced Rodent Model of Autism Spectrum Disorder | Non-high throughput detection technique |
| Amadi CN 2022 | Dietary interventions for autism spectrum disorder: An updated systematic review of human studies | Other types of studies |
| Andreo-Martínez P 2022 | Una propuesta de probiótico basada en el Bifidobacterium para autismo A Probiotic Proposal Based on Bifidobacterium for Autism | Other types of studies |
| Andrusiewicz M 2021 | Commercial microbiota test revealed differences in the composition of Intestinal microorganisms between children with autism spectrum disorders and neurotypical peers | Non-high throughput detection technique |
| Arnold LE 2019 | Probiotics for Gastrointestinal Symptoms and Quality of Life in Autism: A Placebo-Controlled Pilot Trial | No availabel data |
| Avolio E 2022 | Modifications of Behavior and Inflammation in Mice Following Transplant with Fecal Microbiota from Children with Autism | Animal studies |
| Bakir MA 2006 | Bacteroides intestinalis sp. nov., isolated from human faeces | Unrelated studies |
| Barbato P 2012 | Characterization of mucosa-associated and Fecal microbiota of children with austism spectrum disorder | Other types of studies |
| Beopoulos A 2021 | Autonomic Nervous System Neuroanatomical Alterations Could Provoke and Maintain Gastrointestinal Dysbiosis in Autism Spectrum Disorder (ASD): A Novel Microbiome–Host Interaction Mechanistic Hypothesis | Other types of studies |
| Bermudez-Martin P 2021 | The microbial metabolite p-Cresol induces autistic-like behaviors in mice by remodeling the gut microbiota | Animal studies |
| Billeci L 2023 | A randomized controlled trial into the effects of probiotics on electroencephalography in preschoolers with autism | Non-high throughput detection technique |
| Bin-Khattaf RM 2022 | Probiotic Ameliorating Effects of Altered GABA/Glutamate Signaling in a Rodent Model of Autism | Non-high throughput detection technique |
| Bloemendaal M 2023 | The role of the gut microbiota in patients with Kleefstra syndrome | Unrelated studies |
| Boktor JC 2022 | Global metabolic profiles in a non-human primate model of maternal immune activation: implications for neurodevelopmental disorders | No availabel data |
| Bonet ME 2021 | Prebiotic effect of yacon (Smallanthus sonchifolius) on intestinal mucosa using a mouse model | No availabel data |
| Buffington SA 2016 | Microbial Reconstitution Reverses Maternal Diet Induced Social and Synaptic Deficits in Offspring | Animal studies |
| Campbell AS 2022 | Safety and target engagement of an oral small-molecule sequestrant in adolescents with autism spectrum disorder: an open-label phase 1b/2a trial | Non-high throughput detection technique |
| Cao L 2022 | Food Allergy-Induced Autism-Like Behavior is Associated with Gut Microbiota and Brain mTOR Signaling | Animal studies |
| Casas DA 2020 | Dysbiosis in a triplet with an autism spectrum disorder: A case study | Other types of studies |
| Chen K 2020 | Therapeutic Effects of the In Vitro Cultured Human Gut Microbiota as Transplants on Altering Gut Microbiota and Improving Symptoms Associated with Autism Spectrum Disorder | Animal studies |
| Chen K 2023 | The histone H3K4 demethylase KDM5 modulate gut microbiota composition to affect social behavior | Other types of studies |
| Chen Y 2020 | Gut Bacteria Shared by Children and Their Mothers Associate with Developmental Level and Social Deficits in Autism Spectrum Disorder | No availabel data |
| Chen Y 2020 | FTACMT study protocol: a multicentre, double- blind, randomised, placebo- controlled trial of faecal microbiota transplantation for autism spectrum disorder | No available control group |
| Chen Y 2021 | Fecal microbiota transplantation for children with ASD: a multicenter, randomized, double-blind controlled study | Non-high throughput detection technique |
| Chrisman BS 2021 | Improved detection of disease‑associated gut microbes using 16S sequence‑based biomarkers | Unrelated studies |
| Clancy AK 2021 | Antibiotics followed by an ultra filtrate Fecal microbiota transplantation improves symptoms of autism in an adult male | Other types of studies |
| Coretti L 2017 | Sex-related alterations of gut microbiota composition in the BTBR mouse model of autism spectrum disorder | Animal studies |
| Coretti L 2018 | Gut Microbiota Features in Young Children With Autism Spectrum Disorders | Duplicates studies |
| Cristiano C 2018 | Palmitoylethanolamide counteracts autistic-like behaviours in BTBR T+tf/J mice: Contribution of central and peripheral mechanisms | Animal studies |
| Deng W 2022 | Metformin Alleviates Autistic-Like Behaviors Elicited by High-Fat Diet Consumption and Modulates the Crosstalk Between Serotonin and Gut Microbiota in Mouse | Animal studies |
| Dinesh KS 2022 | An Open Label Randomized Control Trial to Assess the Impact of Ayurveda Lifestyle Guidelines and Polyherbal Compounds in Bacterial Flora W.S.R to E. coli and Shigella in Children with Autism Spectrum Disorder | Unrelated studies |
| Doenyas C 2022 | Potential Role of Epigenetics and Redox Signaling in the Gut-Brain Communication and the Case of Autism Spectrum Disorder | Non-high throughput detection technique |
| Dong T 2020 | Prenatal exposure to glufosinate ammonium disturbs gut microbiome and induces behavioral abnormalities in mice | Animal studies |
| Dooling SW 2022 | The Effect of Limosilactobacillus reuteri on Social Behavior Is Independent of the Adaptive Immune System | Non-high throughput detection technique |
| Dovgan K 2023 | Bidirectional relationship between internalizing symptoms and gastrointestinal problems in youth with Autism Spectrum Disorder | Non-high throughput detection technique |
| Dubourdieu MPM 2019 | Relationship between nutritional status and Intestinal microbiota of school age children with autism spectrum disorder | Other types of studies |
| Faruqui NA 2021 | Gut microorganisms and neurological disease perspectives | Other types of studies |
| Franco C 2022 | Impairment in the Intestinal Morphology and in the Immunopositivity of Toll-like Receptor-4 and Other Proteins in an Autistic Mouse Model | Non-high throughput detection technique |
| Galova E 2022 | Characteristics of Intestinal Microbiome in Children with Autism | Non-high throughput detection technique |
| Galova E 2022 | Intestinal Microbiome Shifts in Children with Different Severity of Autism | Non-high throughput detection technique |
| Golubeva AV 2017 | Microbiota-related Changes in Bile Acid & Tryptophan Metabolism are Associated with Gastrointestinal Dysfunction in a Mouse Model of Autism | Animal studies |
| Gondalia SV 2012 | Molecular Characterisation of Gastrointestinal Microbiota of Children With Autism (With and Without Gastrointestinal Dysfunction) and Their Neurotypical Siblings | Negative results |
| Grimaldi R 2018 | A prebiotic intervention study in children with autism spectrum disorders (ASDs) | No available control group |
| Gu Y 2022 | Correlation among gut microbiota; fecal metabolites and autism-like behavior in an adolescent valproic acid-induced rat autism model | Animal studies |
| Gu YY 2021 | Sex-specific differences in the gut microbiota and fecal metabolites in an adolescent valproic acid-induced rat autism model | Animal studies |
| Guiducci L 2022 | Vitamin D Status in Children with Autism Spectrum Disorders: Determinants and Effects of the Response to Probiotic Supplementation | Non-high throughput detection technique |
| Hong RP 2022 | The Difference of Gut Microbiota and Their Correlations With Urinary Organic Acids Between Autistic Children With and Without Atopic Dermatitis | No available control group |
| Hsiao EY 2013 | Microbiota modulate behavioral and physiological abnormalities associated with neurodevelopmental disorders | Animal studies |
| Huang M 2021 | Microbiome-Specific Statistical Modeling Identifies Interplay Between GastroIntestinal Microbiome and Neurobehavioral Outcomes in Patients With Autism: A Case Control Study | No availabel data |
| Huh J 2021 | GUT-brain axis | Other types of studies |
| Inoue R 2019 | Dietary supplementation with partially hydrolyzed guar gum helps improve constipation and gut dysbiosis symptoms and behavioral irritability in children with autism spectrum disorder | Unrelated studies |
| Jensen MH 2022 | An autism spectrum disorder-related risk gene impacts gut tissue mechanics and the gut microbiome in Drosophila melanogaster | Other types of studies |
| Kałużna-Czaplińska J 2012 | The level of arabinitol in autistic children after probiotic therapy | Non-high throughput detection technique |
| Kang DW 2019 | Long-term benefit of Microbiota Transfer Therapy on autism symptoms and gut microbiota | No available control group |
| Karnachuk OV 2021 | Desulfovibrio desulfuricans A Y5 Isolated from a Patient with Autism Spectrum Disorder Binds Iron in Low-Soluble Greigite and Pyrite | Unrelated studies |
| Kim E 2022 | Maternal gut bacteria drives intestinal inflammation in offspring with neurodevelopmental disorders by altering the chromatin landscape of CD4+ T cells | No availabel data |
| Kim JI 2022 | The mediating role of the gut microbiome in the association between ambient air pollution and autistic traits | No available control group |
| Kong Q 2021 | Daily intake of Lactobacillus alleviates autistic-like behaviors by ameliorating the 5-hydroxytryptamine metabolic disorder in VPA-treated rats during weaning and sexual maturation | Animal studies |
| Kong Q 2021 | The autistic-like behaviors development during weaning and sexual maturation in VPA-induced autistic-like rats is accompanied by gut microbiota dysbiosis | Animal studies |
| Kong Q 2022 | Bifidobacterium longum CCFM1077 Ameliorated Neurotransmitter Disorder and Neuroinflammation Closely Linked to Regulation in the Kynurenine Pathway of Autistic-like Rats | Animal studies |
| Kong X 2021 | Altered Autonomic Functions and Gut Microbiome in Individuals with Autism Spectrum Disorder (ASD): Implications for Assisting ASD Screening and Diagnosis | No availabel data |
| Kong XJ 2021 | Probiotic and Oxytocin Combination Therapy in Patients with Autism Spectrum Disorder: A Randomized, Double-Blinded, placebo controlled pilot trial | No available control group |
| Kushak RI 2017 | Analysis of the duodenal microbiome in children with autism | Other types of studies |
| Laghi L 2021 | Are Fecal Metabolome and Microbiota Profiles Correlated with Autism Severity? A Cross-Sectional Study on ASD Preschoolers | No available control group |
| Lee GA 2021 | Maternal Immune Activation Causes Social Behavior Deficits and Hypomyelination in Male Rat Offspring with an Autism-Like Microbiota Profile | Animal studies |
| Li J 2022 | Congenitally underdeveloped intestine drives autism-related gut microbiota and behavior | Animal studies |
| LI N 2019 | Fecal bacteria transplantation relieves autistic behavior in children with autism by improving Intestinal barrier disruption | Other types of studies |
| Li Y 2020 | The gut microbiota regulates autism-like behavior by mediating vitamin B6 homeostasis in EphB6-deficient mice | Animal studies |
| Lim JS 2017 | Modeling environmental risk factors of autism in mice induces IBD-related gut microbial dysbiosis and hyperserotonemia | Animal studies |
| Lin T 2022 | Amelioration of Maternal Immune Activation-Induced Autism Relevant Behaviors by Gut Commensal Parabacteroides goldsteiniiby Gut Commensal Parabacteroides goldsteinii | Non-high throughput detection technique |
| Liu D 2023 | Intestinal metabolites and the risk of autistic spectrum disorder: A two-sample Mendelian randomization study | Non-high throughput detection technique |
| Liu F 2018 | The valproic acid rat model of autism presents with gut bacterial dysbiosis similar to that in human autism | Animal studies |
| Liu G 2022 | Gut dysbiosis impairs hippocampal plasticity and behaviors by remodeling serum metabolome | Animal studies |
| Liu J 2017 | Effect of vitamin A supplementation on gut microbiota in children with autism spectrum disorders - a pilot study | No available control group |
| Liu J 2022 | GW4064 Alters Gut Microbiota Composition and Counteracts Autism-Associated Behaviors in BTBR T+tf/J Mice | Animal studies |
| Liu X 2022 | Rescue of social deficits by early-life melatonin supplementation through modulation of gut microbiota in a murine model of autism | Animal studies |
| Luna RA 2016 | Distinct Microbiome-Neuroimmune Signatures Correlate WithВ Functional Abdominal Pain in Children With Autism Spectrum Disorder | Other types of studies |
| Meguid NA 2022 | Molecular Characterization of Probiotics and Their Influence on Children with Autism Spectrum Disorder | Non-high throughput detection technique |
| Mensi MM 2021 | Lactobacillus plantarum PS128 and Other Probiotics in Children and Adolescents with Autism Spectrum Disorder: A Real-World Experience | Non-high throughput detection technique |
| Mintál K 2022 | Novel probiotic treatment of autism spectrum disorder associated social behavioral symptoms in two rodent models | No availabel data |
| Mohammad FK 2022 | A Computational Framework for Studying Gut-Brain Axis in Autism Spectrum Disorder | Unrelated studies |
| Nankova BB 2014 | Enteric Bacterial Metabolites Propionic and Butyric Acid Modulate Gene Expression, Including CREB-Dependent Catecholaminergic Neurotransmission, in PC12 Cells -Possible Relevance to Autism Spectrum Disorders | Non-high throughput detection technique |
| Navalón P 2022 | Study Protocol: Gut microbiota profiles implicated in the onset of autism spectrum disorders in preterm infants: A two-year follow-up study | Unrelated studies |
| Nettleton JE 2021 | Prebiotic; Probiotic; and Synbiotic Consumption Alter Behavioral V ariables and Intestinal Permeability and Microbiota in BTBR Mouse | Animal studies |
| Ni J 2022 | Gut Microbiota and Psychiatric Disorders: A Two-Sample Mendelian Randomization Study | Unrelated studies |
| Noto A 2017 | Urinary metabolome in autistic children and in their unaffected siblings: preliminary data on the role of oxidative stress and gut dysbiosis | Other types of studies |
| Oroojzadeh P 2022 | Psychobiotics: the Influence of Gut Microbiota on the Gut-Brain Axis in Neurological Disorders | Non-high throughput detection technique |
| Pan ZY 2022 | Beneficial Effects of Repeated Washed Microbiota Transplantation in Children With Autism | Non-high throughput detection technique |
| Picardi A 2012 | Gut permeability, faecal microflora in the autism spectrum disorders. | Other types of studies |
| Pietrucci D 2022 | Machine Learning Data Analysis Highlights the Role of Parasutterella and Alloprevotella in Autism Spectrum Disorders | Unrelated studies |
| Pochakom A 2022 | Selective Probiotic T reatment Positively Modulates the Microbiota–Gut–Brain Axis in the BTBR Mouse Model of Autism | Animal studies |
| Pu Y 2020 | Maternal glyphosate exposure causes autism-like behaviors in offspring through increased expression of soluble epoxide hydrolase | Animal studies |
| Qi Z 2021 | A Novel and Reliable Rat Model of Autism | Animal studies |
| Ramani P 2021 | Can probiotics benefit young people with autism spectrum disorders? | Other types of studies |
| Sanctuary MR 2019 | Pilot study of probiotic/colostrum supplementation on gut function in children with autism and gastrointestinal symptoms | Unrelated studies |
| Santocchi E 2016 | Gut to brain interaction in Autism Spectrum Disorders: a randomized controlled trial on the role of probiotics on clinical, biochemical and neurophysiological parameters | Non-high throughput detection technique |
| Sauer AK 2019 | Altered Intestinal Morphology and Microbiota Composition in the Autism Spectrum Disorders Associated SHANK3 Mouse Model | Animal studies |
| Sauer AK 2019 | Association of zinc deficiency with gastro-intestinal abnormalities in an autism spectrum disorder mouse model | Other types of studies |
| Sauer AK 2021 | Zinc is a key regulator of gastrointestinal development; microbiota composition and inflammation with relevance for autism spectrum disorders | Non-high throughput detection technique |
| Sen P 2022 | The live biotherapeutic Blautia stercoris MRx0006 attenuates social deficits, repetitive behaviour, and anxiety-like behaviour in a mouse model relevant to autism | Non-high throughput detection technique |
| Septyaningtrias DE 2020 | Altered microbiota composition reflects enhanced communication in 15q11-13 CNV mice | Animal studies |
| Serra D 2022 | Attenuation of Autism-like Behaviors by an Anthocyanin-Rich Extract from Portuguese Blueberries via Microbiota–Gut–Brain Axis Modulation in a V alproic Acid Mouse Model | Animal studies |
| Sgritta M 2019 | Mechanisms Underlying Microbial-Mediated Changes in Social Behavior in Mouse Models of Autism Spectrum Disorder | Animal studies |
| Sharon G 2019 | Human Gut Microbiota from Autism Spectrum Disorder Promote Behavioral Symptoms in Mice | Animal studies |
| Smieško G 2021 | Probiotics and fecal bacteriotherapy: the line between deception and treatment | Non-high throughput detection technique |
| Smith CJ 2019 | Combined exposure to air pollution and maternal stress induces sex-specific, autism-like social behavior deficits and gut dysbiosis in mice | Other types of studies |
| Son JS 2015 | Comparison of Fecal Microbiota in Children with Autism Spectrum Disorders and Neurotypical Siblings in the Simons Simplex Collection | Negative results |
| Srinivasjois R 2015 | Probiotic supplementation in children with autism spectrum disorder | Other types of studies |
| Sunand K 2021 | Synbiotic effect of probiotic complex with polyphenols combination for treatment of valproic acid-induced prenatal model of autism | Other types of studies |
| Tabouy L 2018 | Dysbiosis of microbiome and probiotic treatment in a genetic model of autism spectrum disorders | Animal studies |
| Tartaglione A M 2022 | Maternal immune activation induces autism-like changes in behavior; neuroinflammatory profile and gut microbiota in mouse offspring of both sexes | Animal studies |
| Theije CGM 2014 | Altered gut microbiota and activity in a murine model of autism spectrum disorders | Animal studies |
| Tonkaz GY 2023 | Determinants of Leaky Gut and Gut Microbiota Differences in Children With Autism Spectrum Disorder and Their Siblings | Non-high throughput detection technique |
| Urbonas Y 2018 | Fecal transplantation for autism spectrum disorders | Other types of studies |
| Wang J 2020 | Effects of Dietary Fat Profile on Gut microbiota in Valproate Animal Model of Autism | Animal studies |
| Wang J 2023 | Fecal microbiota transplantation improves VPA-induced ASD mice by modulating the serotonergic and glutamatergic synapse signaling pathways | Animal studies |
| Wang X 2019 | Oral probiotic administration during pregnancy prevents autism-related behaviors in offspring induced by maternal immune activation via anti-inflammation in mice | Non-high throughput detection technique |
| Way H 2019 | Investigating the ASD IBS microbiome | Other types of studies |
| Wouw M 2021 | Kefir ameliorates specific microbiota-gut-brain axis impairments in a mouse model relevant to autism spectrum disorder | Animal studies |
| Xiao L 2021 | Fecal Microbiome Transplantation from Children with Autism Spectrum Disorder Modulates Tryptophan and Serotonergic Synapse Metabolism and Induces Altered Behaviors in Germ Free Mice | Animal studies |
| Xu Y 2022 | Leveraging Existing 16SrRNA Microbial Data to Define a Composite Biomarker for Autism Spectrum Disorder | Other types of studies |
| Yang X 2022 | Effect of stigma maydis polysaccharide on the gut microbiota and transcriptome of VPA induced autism model rats | Animal studies |
| Yu Y 2022 | Changes to gut amino acid transporters and microbiome associated with increased E/I ratio in Chd8+/− mouse model of ASD-like behavior | Animal studies |
| Yu Y 2022 | Metabolic and Proteomic Profiles Reveal the Response of the ASD-Associated Resistant Strain 6-1 of Lactobacillus plantarum to Propionic Acid | Non-high throughput detection technique |
| Zafar U 2021 | The Link Between Autism Spectrum Disorder And Gastrointestinal Microbiota | Other types of studies |
| Zeng J 2022 | Hematopoietic stem cell transplantation ameliorates maternal diabetes–mediated gastrointestinal symptoms and autism-like behavior in mouse offspring | Animal studies |
| Zhang L 2022 | The role of probiotics in children with autism spectrum disorders: A study protocol for a randomised controlled trial | Non-high throughput detection technique |
| Zhang W 2022 | Lactobacillus reuteri normalizes altered fear memory in male Cntnap4 knockout mice | Animal studies |
| Zhang Y 2022 | Lactiplantibacillus plantarum ST-III-fermented milk improves autistic-like behaviors in valproic acid-induced autism spectrum disorder mice by altering gut microbiota | Animal studies |
| Zhang Y 2022 | Effects of Washed Fecal Bacteria Transplantation in Sleep Quality, Stool Features and Autism Symptomatology | No availabel data |
| Zhao R 2019 | Correlation between gut microbiota and behavior symptoms in children with autism spectrum disorder | Study in other languages |
| Zhu J 2022 | Alterations in Gut Vitamin and Amino Acid Metabolism are Associated with Symptoms and Neurodevelopment in Children with Autism Spectrum Disorder | No availabel data |
| Zoccante L 2022 | The "Connectivome Theory": A New Model to Understand Autism Spectrum Disorders | Non-high throughput detection technique |
| Zurita MF 2020 | Analysis of gut microbiome, nutrition and immune status in autism spectrum disorder: a case-control study in Ecuador | No availabel data |

**Table S3.** Quality assessment of studies included assessed by the Newcastle Ottawa Scale.

| **Study name** | **Selection** | | | | **Comparability** | **Exposure** | | | **Total** |
| --- | --- | --- | --- | --- | --- | --- | --- | --- | --- |
|  | **Is the case definition adequate?** | **Representativeness of the cases** | **Selection of Controls** | **Definition of Controls** | **Comparability of cases and controls on the basis of the design or analysis** | **Ascertainment of exposure** | **Same method of ascertainment for cases and controls** | **Non-Response rate** |  |
| Agarwala S 2021(1) | * | * | * | * | ** | * | * | * | 9 |
| Angelis MD 2013(2) | * | * | * | * | ** | * | * | * | 9 |
| Averina OV 2020(3) | * | * | * | * | *- | * | * | * | 8 |
| Berding K 2018(4) | - | * | * | * | *- | * | * | * | 8 |
| Berding K 2020(5) | - | * | * | * | *- | * | * | * | 8 |
| Cao X 2021(6) | * | * | - | * | *- | * | * | * | 7 |
| Chen Y 2022(7) | * | * | * | * | ** | * | * | * | 9 |
| Chen Z 2021(8) | * | * | * | * | *- | * | * | * | 8 |
| Claudia C 2019(9) | * | * | * | * | *- | * | * | * | 8 |
| Coretti L 2018(10) | * | * | * | * | *- | * | * | * | 8 |
| Dan Z 2020(11) | * | * | - | * | *- | * | * | * | 7 |
| David MM 2021(12) | * | * | * | * | ** | * | * | * | 9 |
| Dilmore A H 2021(13) | - | * | - | * | -- | * | * | * | 5 |
| Ding H 2021(14) | * | * | - | * | *- | * | * | * | 7 |
| Ding X 2020(15) | * | * | - | * | ** | * | * | * | 9 |
| Finegold SM 2010(16) | * | * | * | * | *- | * | * | * | 8 |
| Fu SC 2021(17) | * | - | - | * | *- | * | * | * | 6 |
| Fujishiro S 2022(18) | * | * | * | * | ** | * | * | * | 9 |
| Galova E 2022(19) | * | - | - | * | -- | * | * | * | 6 |
| Galova E 2022(20) | * | - | - | * | -- | * | * | * | 6 |
| Ha S 2021(21) | * | * | * | * | *- | * | * | * | 8 |
| Huang M 2021(22) | * | * | - | * | ** | * | * | * | 8 |
| Huang M 2021(23) | * | * | - | * | *- | * | * | * | 7 |
| Inoue R 2016(24) | * | - | * | * | *- | * | * | * | 7 |
| Jennifer F 2021(25) | * | * | * | * | ** | * | * | * | 9 |
| Jin J 2019(26) | * | * | - | * | ** | * | * | * | 8 |
| Kang DW 2013(27) | * | - | - | * | *- | * | * | * | 6 |
| Kang DW 2017(28) | * | - | - | * | *- | * | * | * | 6 |
| Kang DW 2017(29) | * | * | - | * | *- | * | * | * | 7 |
| Kong X 2019(30) | * | * | - | * | *- | * | * | * | 7 |
| Kovtun AS 2020(31) | * | * | * | * | * | * | * | * | 9 |
| Kushak RI 2017(32) | * | - | * | * | *- | * | * | * | 7 |
| Levi Mortera S 2022(33) | * | * | * | * | ** | * | * | * | 9 |
| Li J 2022(34) | * | * | * | * | ** | * | * | * | 9 |
| Li N 2019(35) | * | * | * | * | ** | * | * | * | 9 |
| Liu S 2019(36) | * | * | - | * | *- | * | * | * | 7 |
| Lou M 2022(37) | * | * | * | * | *- | * | * | * | 9 |
| Luna RA 2017(38) | * | * | * | * | ** | * | * | * | 9 |
| Maigoro AY 2021(39) | * | * | * | * | *- | * | * | - | 7 |
| Nirmalkar K 2022(40) | * | * | - | * | *- | * | * | * | 7 |
| Niu M 2019(41) | * | * | * | * | ** | * | * | * | 9 |
| Plaza-Díaz J 2019(42) | * | * | * | * | ** | * | * | * | 9 |
| Pulikkan J 2018(43) | * | * | * | * | ** | * | * | * | 9 |
| Rose DR 2018(44) | * | * | * | * | ** | * | * | * | 9 |
| Sgritta M 2019(45) | * | * | - | * | *- | * | * | * | 7 |
| Strati F 2017(46) | * | * | - | * | *- | * | * | * | 7 |
| Sun H 2019(47) | * | * | - | * | *- | * | * | * | 7 |
| Tomova A 2020(48) | * | * | - | * | *- | * | * | * | 7 |
| Tomova A 2020(49) | * | * | - | * | *- | * | * | * | 7 |
| Tong C 2022(50) | * | * | - | * | *- | * | * | * | 7 |
| Vernocchi P 2022(51) | * | * | - | * | *- | * | * | * | 7 |
| Wan Y 2022(52) | * | * | * | * | *- | * | * | * | 8 |
| Wang M 2019(53) | * | * | * | * | *- | * | * | * | 8 |
| Wang Y 2020(54) | * | * | - | * | *- | * | * | * | 7 |
| Williams BL 2011(55) | * | * | * | * | ** | * | * | * | 9 |
| Wong OWH 2022(56) | * | * | - | * | *- | * | * | * | 7 |
| Xie X 2022(57) | * | * | - | * | ** | * | * | * | 8 |
| Ye F 2021(58) | * | * | * | * | ** | * | * | * | 9 |
| Yu R 2022(59) | * | * | - | * | -- | * | * | * | 6 |
| Zhai Q 2019(60) | * | - | - | * | *- | * | * | * | 7 |
| Zhang M 2018(61) | * | - | - | * | *- | * | * | * | 6 |
| Zhang Q 2021(62) | * | * | - | * | *- | * | * | * | 7 |
| Zhang Y 2020(63) | * | * | - | * | ** | * | * | * | 8 |
| Zou R 2020(64) | * | * | - | - | *- | * | * | * | 6 |

Note: A study can be awarded a maximum of one star for each numbered item within the Selection and Exposure categories. A maximum of two stars can be given for Comparability

**Table S4.** Detailed information for each study.

| **Study** | **Country** | **Sample size Patients Control** | **Age** | **Sex (male/female)** | **BMI** | **Intervention** | **Diagnostic criteria** | **CARS (mean ± SD)** | **Sample** | **Sequencing method** | **Amplicon region** | **Comparison groups** |
| --- | --- | --- | --- | --- | --- | --- | --- | --- | --- | --- | --- | --- |
| Agarwala S 2021(1) | India | n=30 n=30 | - | - | - | NA | Not reported | - | Fecal | 16S rRNA amplicon sequencing | V4 | ASD vs. control |
| Angelis MD 2013(2) | Italy | n=10 n=10 | - | - | - | NA | DSM-IV-TR | - | Fecal | 16S rRNA amplicon sequencing | V1-V3 | ASD vs. control |
| Averina OV 2020(3) | Russia | Mild-to-moderate ASD n=15 Sever ASD n=21 Control n=21 | Mild-to-moderate ASD 47 years Sever ASD 43 years Control 43 years | Mild-to-moderate ASD 13/2 Sever ASD 17/4 Control 14/7 | - | NA | DSM-V | Mild-to-moderate ASD 36.0 Sever ASD 47.7 | Fecal | Metagenomic sequencing | - | ASD vs. control |
| Berding K 2018(4) | USA | n=26 n=32 | 4.1 ± 1.6 years 4.8 ± 1.8 years | 19/7 19/13 | - | NA | Not reported | - | Fecal | 16S rRNA amplicon sequencing | V3-V4 | ASD vs. control |
| Berding K 2020(5) | USA | n=26 n=32 | 4.1 ± 1.6 years 4.8 ± 1.8 years | 19/7 19/13 | - | NA | Clinical diagnosis | - | Fecal | 16S rRNA amplicon sequencing | V3-V4 | ASD vs. control |
| Cao X 2021(6) | China | n=45 n=41 | 6.8 ± 3.8 years 5.2 ± 1.0 years | 36/9 34/7 | 16.55 ± 2.43 15.17 ± 1.70 | NA | DSM-V | 36.7 ± 4.6 | Fecal | 16S rRNA amplicon sequencing | V4 | ASD vs. control |
| Chen Y 2022(7) | China | n=82 n=31 | 17.2 ± 4.9 years 13.0 ± 3.9 years | 82/0 31/0 | 20.86 ± 4.45 17.65 ± 3.33 | NA | DSM-V, ADOS-2, ADI-R | - | Fecal | 16S rRNA amplicon sequencing | V3-V4 | ASD vs. control |
| Chen Z 2021(8) | China | n=62 n=60 | 5.9 ± 1.9 years 6.7 ± 2.2 years | 54/8 27/33 | - | NA | DSM-V | 32.1 ± 3.8 | Fecal | 16S rRNA amplicon sequencing | V3-V4 | ASD vs. control |
| Claudia C 2019(9) | Italy | n=16 n=7 | - | - | - | NA | DSM-V | - | Fecal | Metagenomic sequencing | - | ASD vs. control |
| Coretti L 2018(10) | Italy | n=11 n=14 | 35.0 ± 5.7 years 35.0 ± 8.4 years | 9/2 8/6 | - | NA | DSM-V | - | Fecal | 16S rRNA amplicon sequencing | V3-V4 | ASD vs. control |
| Dan Z 2020(11) | China | n=143 n=143 | 5.2 ± 0.2 years 4.9 ± 0.2 years | 127/16 130/13 | - | NA | DSM-V | - | Fecal | 16S rRNA amplicon sequencing, Metagenomic sequencing | V4 | ASD vs. control |
| David MM 2021(12) | USA | n=60 n=57 | 2-7 years 2-7 years | 43/17 27/22 | - | NA | ADOS, ADI-R | - | Fecal | 16S rRNA amplicon sequencing | V4 | ASD vs. control |
| Dilmore A H 2021(13) | USA | n=39 n=46 | 10.2 ± 8.5 years 39.4 ± 9.8 years | 1/38 3/43 | - | NA | Not reported | - | Fecal | 16S rRNA amplicon sequencing, Metagenomic sequencing | V4 | ASD vs. control |
| Ding H 2021(14) | China | n=25 n=20 | 5.7 ± 1.4 years 5.4 ± 1.8 years | 21/4 12/8 | - | NA | DSM-V | - | Fecal | 16S rRNA amplicon sequencing | V3-V4 | ASD vs. control |
| Ding X 2020(15) | China | n=77 n=50 | 38.5 ± 11.7 years 42.9 ± 14.5 years | 59/18 39/11 | - | NA | DSM-V | 37.2 ± 4.3 | Fecal | 16S rRNA amplicon sequencing | V4 | ASD vs. control |
| Finegold SM 2010(16) | USA | n=33 n=8 | - | 24/9 5/3 | - | NA | Clinical diagnosis | - | Fecal | 16S rRNA amplicon sequencing | V1-V2 | ASD vs. control |
| Fu SC 2021(17) | China | n=40 n=40 | 11.1 ± 6.8 years 9.2 ± 7.9 years | 31/9 28/12 | - | NA | DSM-V | - | Fecal | 16S rRNA amplicon sequencing | - | ASD vs. control |
| Fujishiro S 2022(18) | Japan | n=7 n=9 | 10.1 years 14.6 years | 5/2 6/3 | - | NA | Denver II, ASSQ, M-CHAT | - | Fecal | 16S rRNA amplicon sequencing | V1-v4, v6, v7 | ASD vs. control |
| Galova E 2022(19) | Russia | n=46 n=20 | 4.9 ± 1.6 years 5.1 ± 1.2 years | - | - | NA | Not reported | - | - | - | - | ASD vs. control |
| Galova E 2022(20) | Russia | n=46 n=20 | 4.9 ± 1.6 years 5.1 ± 1.2 years | - | - | NA | Not reported | - | - | - | - | ASD vs. control |
| Ha S 2021(21) | Korea | n=54 n=39 | 7.0 ± 2.1 years 6.0 ± 1.7 years | 43/11 18/21 | - | NA | DSM-V, ADOS-2, ADI-R, SRS | - | Fecal | 16S rRNA amplicon sequencing | V3-V4 | ASD vs. control |
| Huang M 2021(22) | USA | n=39 n=44 | 4.7 ± 1.1 years 5.1 ± 1.0 years | 32/7 31/13 | - | NA | DSM-V | - | Fecal | 16S rRNA amplicon sequencing | V4–V5 | ASD vs. control |
| Huang M 2021(23) | USA | n=39 n=80 | ASD 4.7 ± 1.1 years First degree relative control 33.9 ± 5.1 years Healthy control 5.1 ± 0.9 years | ASD 32/7 First degree relative control 0/36 Healthy control 31/13 | - | NA | DSM-V | - | Fecal | 16S rRNA amplicon sequencing | V4–V5 | ASD vs. control |
| Inoue R 2016(24) | Japan | n=6 n=6 | - | - | - | NA | DSM-V, PARS, M-CHAT | - | Fecal | 16S rRNA amplicon sequencing | V3-V4 | ASD vs. control |
| Jennifer F 2021(25) | USA | n=49 n=54 | - | 44/5 37/17 | - | NA | ADOS | - | Fecal | 16S rRNA amplicon sequencing | V4 | ASD vs. control |
| Jin J 2019(26) | China | n=45 n=45 | 7. ± 1.2 years 7.3 ± 1.1 years | 39/6 39/6 | - | NA | DSM-V | 36.3 ± 6.3 | Fecal | 16S rRNA amplicon sequencing | V3-V4 | ASD vs. control |
| Kang DW 2013(27) | USA | n=20 n=6 | 6.7 ± 2.7 years 8.3 ± 4.4 years | 18/2 17/3 | - | NA | ADI-R, ADOS, ATEC, PDD-BI | - | Fecal | 16S rRNA amplicon sequencing | V2-V3 | ASD vs. control |
| Kang DW 2017(28) | USA | n=23 n=21 | 8.4 ± 3.4 years 10.1 ± 4.1 years | 22/1 15/6 | - | NA | Not reported | - | Fecal | 16S rRNA amplicon sequencing | V2-V3 | ASD vs. control |
| Kang DW 2017(29) | USA | n=18 n=20 | 10.8 ± 1.6 years 11.4 ± 2.5 years | 16/2 18/2 | 18.2 ± 2.2 17.1 ± 1.1 | NA | ADI-R | - | Fecal | 16S rRNA amplicon sequencing | V4 | ASD vs. control |
| Kong X 2019(30) | USA | n=20 n=19 | 15 years 29 years | 15/5 8/11 | - | NA | DSM-V | - | Fecal | 16S rRNA amplicon sequencing | V3-V4 | ASD vs. control |
| Kovtun AS 2020(31) | Russia | n=30 n=20 | - | - | - | NA | Not reported | - | Fecal | Metagenomic sequencing | - | ASD vs. control |
| Kushak RI 2017(32) | USA | n=21 n=19 | 14.4 ± 1.1 years 16.1 ± 1.3 years | 19/2 10/9 | - | NA | DSM-IV | - | Duodenal mucosa | 16S rRNA amplicon sequencing | - | ASD vs. control |
| Levi Mortera S 2022(33) | Italy | n=10 n=10 | - | - | - | NA | Not reported | - | Fecal | Metagenomic sequencing | - | ASD vs. control |
| Li J 2022(34) | China | n=13 n=12 | 2-7 years 2-7 years | - | - | NA | DSM-V | - | Fecal | 16S rRNA amplicon sequencing | V4 | ASD vs. control |
| Li N 2019(35) | China | n=59 n=30 | 4 years 5 years | 50/9 20/10 | - | NA | DSM-V | - | Fecal | 16S rRNA amplicon sequencing | V1-V2 | ASD vs. control |
| Liu S 2019(36) | China | n=30 n=20 | 4.4 ± 1.5 years 4.3 ± 1.0 years | 25/5 16/4 | - | NA | DSM-V, ICD-10 | - | Fecal | 16S rRNA amplicon sequencing | V3-V4 | ASD vs. control |
| Lou M 2022(37) | China | n=773 n=429 | 16 months - 19 years 11 months - 15 years | - | - | NA | DSM-V | - | Fecal | 16S rRNA amplicon sequencing | V4 | ASD vs. control |
| Luna RA 2017(38) | USA | ASD with gastrointestinal symptoms n=14 ASD with gastrointestinal symptoms n=15 ASD without gastrointestinal symptoms n=6 | ASD with gastrointestinal symptoms 8.5 years Control with gastrointestinal symptoms 10.5 years Control without gastrointestinal symptoms 5.5 years | - | - | NA | ADOS | - | Rectal mucosa | 16S rRNA amplicon sequencing | V1, V3, V4 | ASD with gastrointestinal disorder vs.control |
| Maigoro AY 2021(39) | Korea | n=36 n=21 | - | - | - | NA | Not reported | - | Fecal | Metagenomic sequencing | - | ASD vs. control |
| Nirmalkar K 2022(40) | USA | n=18 n=20 | 7-16 years 7-16 years | - | - | Fecal microbiota transplantation | Not reported | - | Fecal | Metagenomic sequencing | - | ASD treated with microbiota transfer therapy at baseline vs. control |
| Niu M 2019(41) | Italy | n=114 n=40 | 4.5 years 4.2 years | 95/19 20/20 | - | NA | DSM-V | - | Fecal | 16S rRNA amplicon sequencing | - | ASD vs. control |
| Plaza-Díaz J 2019(42) | Spain | n=48 n=57 | 44.2 ± 1.6 months 51 ± 2.6 months | - | 15.86 ± 0.26 16.20 ± 0.20 | NA | ICD-10, DSM-V | 32.7 ± 1.1 | Fecal | 16S rRNA amplicon sequencing | V3-V4 | ASD vs. control |
| Pulikkan J 2018(43) | India | n=30 n=24 | 9.5 years 9.5 years | 28/2 15/9 | 14.78 15.79 | NA | CARS, DSM-V, ISAA | > 36.5 | Fecal | 16S rRNA amplicon sequencing | V3 | ASD vs. control |
| Rose DR 2018(44) | USA | n=50 n=41 | - | - | - | NA | DSM-IV | - | Fecal | 16S rRNA amplicon sequencing | V3-V4 | ASD with gastrointestinal symptoms vs. control with gastrointestinal symptoms |
| Sgritta M 2019(45) | France | n=38 n=15 | 33 ± 1 years 28 ± 2 years | 26/17 10/5 | - | NA | DSM-IV R, ADOS-R | - | Fecal | 16S rRNA amplicon sequencing | V3–V4 | ASD vs. control |
| Strati F 2017(46) | Italy | n=40 n=40 | 10 years 7 years | 31/9 28/12 | - | NA | DSM-V | 47 | Fecal | 16S rRNA amplicon sequencing | V3-V5 | ASD vs. control |
| Sun H 2019(47) | China | n=9 n=6 | - | 8/1 4/2 | - | NA | ICD-11 | - | Fecal | 16S rRNA amplicon sequencing | V3-V4 | ASD vs. control |
| Tomova A 2020(48) | Slovakia | n=63 n=27 | 5.0 ± 0.2 years 5.6 ± 0.4 years | 63/0 27/0 | - | NA | DSM-V | - | Fecal | 16S rRNA amplicon sequencing | V1-V9 | ASD vs. control |
| Tomova A 2020(49) | Slovakia | n=46 n=16 | 6.3 ± 1.5 years 5.1 ± 1.7 years | 46/0 16/0 | 17.1 ± 3.7 16.2 ± 2.0 | NA | DSM-V | - | Fecal | 16S rRNA amplicon sequencing | V1-V9 | ASD vs. control |
| Tong C 2022(50) | China | n=26 n=26 | 4.1 ± 1.0 years 4.0 ± 0.9 years | 23/3 23/3 | - | NA | DSM-V | 32.9 ± 8.6 | Fecal | Metagenomic sequencing | - | ASD vs. control |
| Vernocchi P 2022(51) | Italy | n=41 n=35 | 6.5 ± 3.4 years 8.0 ± 3.6 years | 36/5 21/14 | 16.50 ± 3.17  15.84 ± 1.85 | NA | DSM-V, ADOS-2, ADI-R | - | Fecal | 16S rRNA amplicon sequencing | V3–V4 | ASD vs. control |
| Wan Y 2022(52) | China | n=64 n=64 | 59 months 56 months | 53/11 54/10 | - | NA | DSM-IV, DSM-V | - | Fecal | Metagenomic sequencing | - | ASD vs. control |
| Wang M 2019(53) | China | n=43 n=31 | 4.5 ± 2.2 years 4.9 ± 1.0 years | 36/7 31/27 | - | NA | DSM-V | - | Fecal | Metagenomic sequencing | - | ASD vs. control |
| Wang Y 2020(54) | China | n=26 n=24 | 4.3 years 4.6 years | 24/2 22/2 | - | NA | DSM-V, CCMD-3 | - | Fecal | 16S rRNA amplicon sequencing | V1-V2 | ASD vs. control |
| Williams BL 2011(55) | USA | n=15 n=7 | 4.5 ± 1.3 years 4.0 ± 1.1 years | 15/0 7/0 | - | NA | DSM-IV-TR | - | Ileal and cecal mucosa | 16S rRNA amplicon sequencing | V2 | ASD vs. control |
| Wong OWH 2022(56) | China | ASD with gastrointestinal symptoms 30 ASD without gastrointestinal symptoms 62 Control with gastrointestinal symptoms 32 Control without gastrointestinal symptoms 80 | 8.4 ± 1.5 years 8.4 ± 1.5 years | ASD with gastrointestinal symptoms 30/0 ASD without gastrointestinal symptoms 62/0 Control with gastrointestinal symptoms 32/0 Control without gastrointestinal symptoms 80/0 | ASD with gastrointestinal symptoms 15.670 ± 3.282 ASD without gastrointestinal symptoms 16.730 ± 4.654 Control with gastrointestinal symptoms 17.56 ± 5.07 Control without gastrointestinal symptoms 16.960 ± 4.727 | NA | DSM-V | - | Fecal | 16S rRNA amplicon sequencing | V4 | ASD vs. control |
| Xie X 2022(57) | China | n=101 n=103 | 4.3 ± 1.8 4.4 ± 0.9 | 86/15 80/23 | 16.26 ± 3.67  15.69 ± 2.52 | NA | DSM-V | 35.2 ± 3.9 | Fecal | 16S rRNA amplicon sequencing | V3-V4 | ASD vs. control |
| Ye F 2021(58) | China | n=71 n=18 | 4.3 ± 1.5 years 4.6 ± 1.3 years | 71/0 18/0 | - | NA | DSM-V | - | Fecal | 16S rRNA amplicon sequencing, Metagenomic sequencing | V1-V2 | ASD vs. control |
| Yu R 2022(59) | China | n=24 n=38 | 6.2 years 8.6 years | - | - | NA | DSM-V | - | Fecal | 16S rRNA amplicon sequencing | - | ASD vs. control |
| Zhai Q 2019(60) | China | n=78 n=58 | 5.0 ± 1.0 years 4.9 ± 1.0 years | 56/22 31/27 | - | NA | DSM-IV, ICD-10 | - | Fecal | 16S rRNA amplicon sequencing | V3-V4 | ASD vs. control |
| Zhang M 2018(61) | China | n=35 n=6 | 4.9 ± 1.5 years 4.6 ± 1.1 years | 29/6 5/1 | - | NA | DSM-V | - | Fecal | 16S rRNA amplicon sequencing | V3-V4 | ASD vs. control |
| Zhang Q 2021(62) | China | n=21 n=21 | 17-32 years 17-32 years | 15/6 15/6 | 22.8 35.3 | NA | ICD-10 | - | Fecal | 16S rRNA amplicon sequencing | V3-V4 | ASD vs. obese non-ASD |
| Zhang Y 2020(63) | China | n=14 n=18 | 3.5 ± 1.2 years 4.6 ± 1.1 years | 12/2 13/5 | - | NA | Clinical diagnosis | 40.5 | Fecal | 16S rRNA amplicon sequencing | V3-V4 | ASD vs. control |
| Zou R 2020(64) | China | n=48 n=48 | 5 years 4 years | 38/10 24/24 | 17.4 16.3 | NA | DSM-IV-TR, ADI-R | - | Fecal | 16S rRNA amplicon sequencing | V3-V4 | ASD vs. control |

Abbreviations: BMI, Body Mass Index; CARS, Childhood Autism Rating Scale; ASD, autism spectrum disorder; NA, not applicated; DSM-IV/V/TR, Diagnostic and Statistical Manual of Mental Disorders Fourth/Fifth Edition/Text Revision ; ADOS-2, Autism Diagnostic Observation Schedule Second Edition; ADI-R, Autism Diagnostic Interview-Revised, ASSQ, Autism Spectrum Screening Questionnaire, M-CHAT, Modified Checklist for Autism in Toddlers; ATEC, Autism Treatment Evaluation Checklist; PDD-BI, Pervasive Developmental Disorders Behavior Inventory; ISAA, Infant-Toddler Social and Emotional Assessment; CCMD-3, Chinese Classification and Diagnostic Criteria of Mental Disorders Third Version.

**References**

1. Agarwala S, Naik B, Ramachandra NB. Mucosa-associated specific bacterial species disrupt the intestinal epithelial barrier in the autism phenome. Brain, behavior, & immunity - health. (2021)15:100269. doi: 10.1016/j.bbih.2021.100269

2. De Angelis M, Piccolo M, Vannini L, Siragusa S, De Giacomo A, Serrazzanetti DI, et al. Fecal microbiota and metabolome of children with autism and pervasive developmental disorder not otherwise specified. PloS one. (2013)8:e76993. doi: 10.1371/journal.pone.0076993

3. Averina OV, Kovtun AS, Polyakova SI, Savilova AM, Rebrikov DV, Danilenko VN. The bacterial neurometabolic signature of the gut microbiota of young children with autism spectrum disorders. Journal of medical microbiology. (2020)69:558-71. doi: 10.1099/jmm.0.001178

4. Berding K, Donovan SM. Diet Can Impact Microbiota Composition in Children With Autism Spectrum Disorder. Frontiers in neuroscience. (2018)12:515. doi: 10.3389/fnins.2018.00515

5. Berding K, Donovan SM. Dietary Patterns Impact Temporal Dynamics of Fecal Microbiota Composition in Children With Autism Spectrum Disorder. Frontiers in nutrition. (2019)6:193. doi: 10.3389/fnut.2019.00193

6. Cao X, Liu K, Liu J, Liu YW, Xu L, Wang H, et al. Dysbiotic Gut Microbiota and Dysregulation of Cytokine Profile in Children and Teens With Autism Spectrum Disorder. Frontiers in neuroscience. (2021)15:635925. doi: 10.3389/fnins.2021.635925

7. Chen YC, Lin HY, Chien Y, Tung YH, Ni YH, Gau SS. Altered gut microbiota correlates with behavioral problems but not gastrointestinal symptoms in individuals with autism. Brain, behavior, and immunity. (2022)106:161-78. doi: 10.1016/j.bbi.2022.08.015

8. Chen Z, Shi K, Liu X, Dai Y, Liu Y, Zhang L, et al. Gut Microbial Profile Is Associated With the Severity of Social Impairment and IQ Performance in Children With Autism Spectrum Disorder. Frontiers in psychiatry. (2021)12:789864. doi: 10.3389/fpsyt.2021.789864

9. Carissimi C, Laudadio I, Palone F, Fulci V, Cesi V, Cardona F, et al. Functional analysis of gut microbiota and immunoinflammation in children with autism spectrum disorders. Digestive and liver disease : official journal of the Italian Society of Gastroenterology and the Italian Association for the Study of the Liver. (2019)51:1366-74. doi: 10.1016/j.dld.2019.06.006

10. Coretti L, Paparo L, Riccio MP, Amato F, Cuomo M, Natale A, et al. Gut Microbiota Features in Young Children With Autism Spectrum Disorders. Frontiers in microbiology. (2018)9:3146. doi: 10.3389/fmicb.2018.03146

11. Dan Z, Mao X, Liu Q, Guo M, Zhuang Y, Liu Z, et al. Altered gut microbial profile is associated with abnormal metabolism activity of Autism Spectrum Disorder. Gut microbes. (2020)11:1246-67. doi: 10.1080/19490976.2020.1747329

12. David MM, Tataru C, Daniels J, Schwartz J, Keating J, Hampton-Marcell J, et al. Children with Autism and Their Typically Developing Siblings Differ in Amplicon Sequence Variants and Predicted Functions of Stool-Associated Microbes. mSystems. (2021)6. doi: 10.1128/mSystems.00193-20

13. Dilmore AH, McDonald D, Nguyen TT, Adams JB, Krajmalnik-Brown R, Elijah E, et al. The Fecal Microbiome and Metabolome of Pitt Hopkins Syndrome, a Severe Autism Spectrum Disorder. mSystems. (2021)6:e0100621. doi: 10.1128/mSystems.01006-21

14. Ding H, Yi X, Zhang X, Wang H, Liu H, Mou WW. Imbalance in the Gut Microbiota of Children With Autism Spectrum Disorders. Frontiers in cellular and infection microbiology. (2021)11:572752. doi: 10.3389/fcimb.2021.572752

15. Ding X, Xu Y, Zhang X, Zhang L, Duan G, Song C, et al. Gut microbiota changes in patients with autism spectrum disorders. Journal of psychiatric research. (2020)129:149-59. doi: 10.1016/j.jpsychires.2020.06.032

16. Finegold SM, Dowd SE, Gontcharova V, Liu C, Henley KE, Wolcott RD, et al. Pyrosequencing study of fecal microflora of autistic and control children. Anaerobe. (2010)16:444-53. doi: 10.1016/j.anaerobe.2010.06.008

17. Fu SC, Lee CH, Wang H. Exploring the Association of Autism Spectrum Disorders and Constipation through Analysis of the Gut Microbiome. International journal of environmental research and public health. (2021)18. doi: 10.3390/ijerph18020667

18. Fujishiro S, Tsuji S, Akagawa S, Akagawa Y, Yamanouchi S, Ishizaki Y, et al. Dysbiosis in Gut Microbiota in Children Born Preterm Who Developed Autism Spectrum Disorder: A Pilot Study. (2022):1-9. doi: 10.1007/s10803-022-05682-0

19. Galova E, Shirokova I, Blagonravova A, Vorobyeva O, Martusevich A. Characteristics of Intestinal Microbiome in Children with Autism. Archiveuromedica. (2022)12;1-4. doi: 10.35630/2199-885X/2022/12/1.1

20. Galova E, Martusevich A, Blagonravova A, Vorobyeva O, Popovicheva A. Intestinal Microbiome Shifts in Children with Different Severity of Autism. Archiv EuroMedica. (2022)12;6-7. doi: 10.35630/2199-885X/2022/12/5.5

21. Ha S, Oh D, Lee S, Park J, Ahn J, Choi S, et al. Altered Gut Microbiota in Korean Children with Autism Spectrum Disorders. Nutrients. (2021)13. doi: 10.3390/nu13103300

22. Huang M, Liu K, Wei Z, Feng Z, Chen J, Yang J, et al. Serum Oxytocin Level Correlates With Gut Microbiome Dysbiosis in Children With Autism Spectrum Disorder. Frontiers in neuroscience. (2021)15:721884. doi: 10.3389/fnins.2021.721884

23. Huang M, Liu J, Liu K, Chen J, Wei Z, Feng Z, et al. Microbiome-Specific Statistical Modeling Identifies Interplay Between Gastrointestinal Microbiome and Neurobehavioral Outcomes in Patients With Autism: A Case Control Study. Frontiers in psychiatry. (2021)12:682454. doi: 10.3389/fpsyt.2021.682454

24. Inoue R, Sakaue Y, Sawai C, Sawai T, Ozeki M, Romero-Pérez GA, et al. A preliminary investigation on the relationship between gut microbiota and gene expressions in peripheral mononuclear cells of infants with autism spectrum disorders. Bioscience, biotechnology, and biochemistry. (2016)80:2450-8. doi: 10.1080/09168451.2016.1222267

25. Fouquier J, Moreno Huizar N, Donnelly J, Glickman C, Kang DW, Maldonado J, et al. The Gut Microbiome in Autism: Study-Site Effects and Longitudinal Analysis of Behavior Change. mSystems. (2021)6. doi: 10.1128/mSystems.00848-20

26. Ma B, Liang J, Dai M, Wang J, Luo J, Zhang Z, et al. Altered Gut Microbiota in Chinese Children With Autism Spectrum Disorders. Frontiers in cellular and infection microbiology. (2019)9:40. doi: 10.3389/fcimb.2019.00040

27. Kang DW, Park JG, Ilhan ZE, Wallstrom G, Labaer J, Adams JB, et al. Reduced incidence of Prevotella and other fermenters in intestinal microflora of autistic children. PloS one. (2013)8:e68322. doi: 10.1371/journal.pone.0068322

28. Kang DW, Ilhan ZE, Isern NG, Hoyt DW, Howsmon DP, Shaffer M, et al. Differences in fecal microbial metabolites and microbiota of children with autism spectrum disorders. Anaerobe. (2018)49:121-31. doi: 10.1016/j.anaerobe.2017.12.007

29. Kang DW, Adams JB, Gregory AC, Borody T, Chittick L, Fasano A, et al. Microbiota Transfer Therapy alters gut ecosystem and improves gastrointestinal and autism symptoms: an open-label study. Microbiome. (2017)5:10. doi: 10.1186/s40168-016-0225-7

30. Kong X, Liu J, Cetinbas M, Sadreyev R, Koh M, Huang H, et al. New and Preliminary Evidence on Altered Oral and Gut Microbiota in Individuals with Autism Spectrum Disorder (ASD): Implications for ASD Diagnosis and Subtyping Based on Microbial Biomarkers. Nutrients. (2019)11. doi: 10.3390/nu11092128

31. Kovtun AS, Averina OV, Alekseeva MG, Danilenko VN. Antibiotic Resistance Genes in the Gut Microbiota of Children with Autistic Spectrum Disorder as Possible Predictors of the Disease. Microbial drug resistance (Larchmont, NY). (2020)26:1307-20. doi: 10.1089/mdr.2019.0325

32. Kushak RI, Winter HS, Buie TM, Cox SB, Phillips CD, Ward NL. Analysis of the Duodenal Microbiome in Autistic Individuals: Association With Carbohydrate Digestion. Journal of pediatric gastroenterology and nutrition. (2017)64:e110-e6. doi: 10.1097/mpg.0000000000001458

33. Levi Mortera S, Vernocchi P, Basadonne I, Zandonà A, Chierici M, Durighello M, et al. A metaproteomic-based gut microbiota profiling in children affected by autism spectrum disorders. Journal of proteomics. (2022)251:104407. doi: 10.1016/j.jprot.2021.104407

34. Li J, Wang H, Qing W, Liu F, Zeng N, Wu F, et al. Congenitally underdeveloped intestine drives autism-related gut microbiota and behavior. Brain, behavior, and immunity. (2022)105:15-26. doi: 10.1016/j.bbi.2022.06.006

35. Li N, Yang J, Zhang J, Liang C, Wang Y, Chen B, et al. Correlation of Gut Microbiome Between ASD Children and Mothers and Potential Biomarkers for Risk Assessment. Genomics, proteomics & bioinformatics. (2019)17:26-38. doi: 10.1016/j.gpb.2019.01.002

36. Liu S, Li E, Sun Z, Fu D, Duan G, Jiang M, et al. Altered gut microbiota and short chain fatty acids in Chinese children with autism spectrum disorder. Scientific reports. (2019)9:287. doi: 10.1038/s41598-018-36430-z

37. Lou M, Cao A, Jin C, Mi K, Xiong X, Zeng Z, et al. Deviated and early unsustainable stunted development of gut microbiota in children with autism spectrum disorder. Gut. (2022)71:1588-99. doi: 10.1136/gutjnl-2021-325115

38. Luna RA, Oezguen N, Balderas M, Venkatachalam A, Runge JK, Versalovic J, et al. Distinct Microbiome-Neuroimmune Signatures Correlate With Functional Abdominal Pain in Children With Autism Spectrum Disorder. Cellular and molecular gastroenterology and hepatology. (2017)3:218-30. doi: 10.1016/j.jcmgh.2016.11.008

39. Maigoro AY, Lee S. Gut Microbiome-Based Analysis of Lipid A Biosynthesis in Individuals with Autism Spectrum Disorder: An In Silico Evaluation. Nutrients. (2021)13. doi: 10.3390/nu13020688

40. Nirmalkar K, Qureshi F, Kang DW, Hahn J, Adams JB, Krajmalnik-Brown R. Shotgun Metagenomics Study Suggests Alteration in Sulfur Metabolism and Oxidative Stress in Children with Autism and Improvement after Microbiota Transfer Therapy. International journal of molecular sciences. (2022)23. doi: 10.3390/ijms232113481

41. Niu M, Li Q, Zhang J, Wen F, Dang W, Duan G, et al. Characterization of Intestinal Microbiota and Probiotics Treatment in Children With Autism Spectrum Disorders in China. Frontiers in neurology. (2019)10:1084. doi: 10.3389/fneur.2019.01084

42. Plaza-Díaz J, Gómez-Fernández A, Chueca N, Torre-Aguilar MJ, Gil Á, Perez-Navero JL, et al. Autism Spectrum Disorder (ASD) with and without Mental Regression is Associated with Changes in the Fecal Microbiota. Nutrients. (2019)11. doi: 10.3390/nu11020337

43. Pulikkan J, Maji A, Dhakan DB, Saxena R, Mohan B, Anto MM, et al. Gut Microbial Dysbiosis in Indian Children with Autism Spectrum Disorders. Microbial ecology. (2018)76:1102-14. doi: 10.1007/s00248-018-1176-2

44. Rose DR, Yang H, Serena G, Sturgeon C, Ma B, Careaga M, et al. Differential immune responses and microbiota profiles in children with autism spectrum disorders and co-morbid gastrointestinal symptoms. Brain, behavior, and immunity. (2018)70:354-68. doi: 10.1016/j.bbi.2018.03.025

45. Gonzales J, Marchix J, Aymeric L, Le Berre-Scoul C, Zoppi J, Bordron P, et al. Fecal Supernatant from Adult with Autism Spectrum Disorder Alters Digestive Functions, Intestinal Epithelial Barrier, and Enteric Nervous System. Microorganisms. (2021)9. doi: 10.3390/microorganisms9081723

46. Strati F, Cavalieri D, Albanese D, De Felice C, Donati C, Hayek J, et al. New evidences on the altered gut microbiota in autism spectrum disorders. Microbiome. (2017)5:24. doi: 10.1186/s40168-017-0242-1

47. Sun H, You Z, Jia L, Wang F. Autism spectrum disorder is associated with gut microbiota disorder in children. BMC pediatrics. (2019)19:516. doi: 10.1186/s12887-019-1896-6

48. Tomova A, Soltys K, Repiska G, Palkova L, Filcikova D, Minarik G, et al. Specificity of gut microbiota in children with autism spectrum disorder in Slovakia and its correlation with astrocytes activity marker and specific behavioural patterns. Physiology & behavior. (2020)214:112745. doi: 10.1016/j.physbeh.2019.112745

49. Tomova A, Soltys K, Kemenyova P, Karhanek M, Babinska K. The Influence of Food Intake Specificity in Children with Autism on Gut Microbiota. International journal of molecular sciences. (2020)21. doi: 10.3390/ijms21082797

50. Tong Z, Zhou X, Chu Y, Zhang T, Zhang J, Zhao X, et al. Implications of oral streptococcal bacteriophages in autism spectrum disorder. NPJ biofilms and microbiomes. (2022)8:91. doi: 10.1038/s41522-022-00355-3

51. Vernocchi P, Ristori MV, Guerrera S, Guarrasi V, Conte F, Russo A, et al. Gut Microbiota Ecology and Inferred Functions in Children With ASD Compared to Neurotypical Subjects. Frontiers in microbiology. (2022)13:871086. doi: 10.3389/fmicb.2022.871086

52. Wan Y, Zuo T, Xu Z, Zhang F, Zhan H, Chan D, et al. Underdevelopment of the gut microbiota and bacteria species as non-invasive markers of prediction in children with autism spectrum disorder. Gut. (2022)71:910-8. doi: 10.1136/gutjnl-2020-324015

53. Wang M, Wan J, Rong H, He F, Wang H, Zhou J, et al. Alterations in Gut Glutamate Metabolism Associated with Changes in Gut Microbiota Composition in Children with Autism Spectrum Disorder. mSystems. (2019)4. doi: 10.1128/mSystems.00321-18

54. Wang Y, Li N, Yang JJ, Zhao DM, Chen B, Zhang GQ, et al. Probiotics and fructo-oligosaccharide intervention modulate the microbiota-gut brain axis to improve autism spectrum reducing also the hyper-serotonergic state and the dopamine metabolism disorder. Pharmacological research. (2020)157:104784. doi: 10.1016/j.phrs.2020.104784

55. Williams BL, Hornig M, Buie T, Bauman ML, Cho Paik M, Wick I, et al. Impaired carbohydrate digestion and transport and mucosal dysbiosis in the intestines of children with autism and gastrointestinal disturbances. PloS one. (2011)6:e24585. doi: 10.1371/journal.pone.0024585

56. Wong OWH, Lam AMW, Or BPN, Mo FYM, Shea CKS, Lai KYC, et al. Disentangling the relationship of gut microbiota, functional gastrointestinal disorders and autism: a case-control study on prepubertal Chinese boys. Scientific reports. (2022)12:10659. doi: 10.1038/s41598-022-14785-8

57. Xie X, Li L, Wu X, Hou F, Chen Y, Shi L, et al. Alteration of the fecal microbiota in Chinese children with autism spectrum disorder. Autism research : official journal of the International Society for Autism Research. (2022)15:996-1007. doi: 10.1002/aur.2718

58. Ye F, Gao X, Wang Z, Cao S, Liang G, He D, et al. Comparison of gut microbiota in autism spectrum disorders and neurotypical boys in China: A case-control study. Synthetic and systems biotechnology. (2021)6:120-6. doi: 10.1016/j.synbio.2021.03.003

59. Yu R, Ahmed T, Jiang H, Zhou G, Zhang M, Lv L, et al. Impact of Zinc Oxide Nanoparticles on the Composition of Gut Microbiota in Healthy and Autism Spectrum Disorder Children. Materials (Basel, Switzerland). (2021)14. doi: 10.3390/ma14195488

60. Zhai Q, Cen S, Jiang J, Zhao J, Zhang H, Chen W. Disturbance of trace element and gut microbiota profiles as indicators of autism spectrum disorder: A pilot study of Chinese children. Environmental research. (2019)171:501-9. doi: 10.1016/j.envres.2019.01.060

61. Zhang M, Ma W, Zhang J, He Y, Wang J. Analysis of gut microbiota profiles and microbe-disease associations in children with autism spectrum disorders in China. Scientific reports. (2018)8:13981. doi: 10.1038/s41598-018-32219-2

62. Zhang Q, Zou R, Guo M, Duan M, Li Q, Zheng H. Comparison of gut microbiota between adults with autism spectrum disorder and obese adults. PeerJ. (2021)9:e10946. doi: 10.7717/peerj.10946

63. Zhang Y, Hu N, Cai Q, Zhang F, Zou J, Liu Y, et al. Treatment with the traditional Chinese medicine BuYang HuanWu Tang induces alterations that normalize the microbiome in ASD patients. Bioscience of microbiota, food and health. (2020)39:109-16. doi: 10.12938/bmfh.2019-032

64. Zou R, Xu F, Wang Y, Duan M, Guo M, Zhang Q, et al. Changes in the Gut Microbiota of Children with Autism Spectrum Disorder. Autism research : official journal of the International Society for Autism Research. (2020)13:1614-25. doi: 10.1002/aur.235

**Table S5.** Microbiota diversity in patients with ASD.

| **Study** | **α-diversity assessment** | **α-diversity alteration** | **β-diversity assessment** | **β-diversity alteration** |
| --- | --- | --- | --- | --- |
| Agarwala S 2021 | Shannon | Increased | Bray-Curtis | Significantly different |
| Angelis MD 2013 | Chao1 | Increased | Unweighted Unifrac | Significantly different |
|  | Shannon | Increased |  |  |
| Averina OV 2020 | Shannon | Decreased | NA | NS |
| Berding K 2018 | Chao1 | NS | Weighted UniFrac | Significantly different |
|  | Shannon | NS |  |  |
|  | Simpson | NS | Unweighted UniFrac | Significantly different |
|  | Observed species | NS |  |  |
| Berding K 2020 | Chao1 | NS | Weighted UniFrac  Unweighted UniFrac | NS  NS |
|  | Observed species | NS |  |  |
|  | Shannon | NS |  |  |
| Chen Y 2022 | Shannon | NS | Weighted UniFrac | Significantly different |
|  | Simpson | NS | Unweighted UniFrac  Bray-Curtis | NS  NS |
|  | Faith’s PD | NS |  |  |
|  | Good’s coverage | NS |  |  |
| Chen Z 2021 | Shannon | Decreased | Unweighted UniFrac | NS |
|  | Chao1 | Decreased |  |  |
|  | Observed species | Decreased |  |  |
|  | ACE | Decreased | Bray-Curtis | NS |
|  | Simpson | Decreased |  |  |
|  | InvSimpson | Decreased |  |  |
| Claudia C 2019 | Fisher | Decreased | NA | NS |
| Coretti L 2018 | Shannon | Increased | Weighted UniFrac  Unweighted UniFrac | Significantly different  Significantly different |
|  | Good’s coverage | NA |  |  |
|  | Observed species | NA |  |  |
| Dan Z 2020 | Observed species | Decreased | Weighted UniFrac | Significantly different |
|  |  |  | Unweighted UniFrac | Significantly different |
|  |  |  | Bray-Curtis | Significantly different |
| David MM 2021 | Faith’s PD | NS | NA | NS |
|  | Shannon | NS |  |  |
| Dilmore A H 2021 | Shannon | NS | Unweighted UniFrac | Significantly different |
|  | Chao | NS |  |  |
|  | ACE | NS |  |  |
| Ding X 2020 | Observed species | Increased | Unweighted UniFrac  Bray-Curtis | Significantly different  Significantly different |
|  | Chao1 | Increased |  |  |
|  | Shannon | Increased |  |  |
| Finegold SM 2010 | ACE | Increased | NA | Significantly different |
|  | Chao1 | Increased |  |  |
| Fujishiro S 2022 | Shannon | Increased | Bray–Curtis | Significantly different |
|  | Simpson | Increased |  |  |
|  | Observed species | NS |  |  |
| Huang M 2021 | Shannon | NS | Weighted UniFrac | NS |
|  |  |  | Unweighted UniFrac | NS |
|  |  |  | Bray-Curtis | NS |
| Jin J 2019 | ACE | Decreased | Unweighted UniFrac | Significantly different |
|  | Faith’s PD | Decreased | Bray-Curtis | Significantly different |
| Kang DW 2013 | Chao1 | Decreased | NA | NS |
|  | Faith’s PD | Decreased |  |  |
|  | Observed species | NA |  |  |
|  | Shannon | NA |  |  |
| Kang DW 2017 | Observed species | Decreased | Weighted UniFrac | Significantly different |
|  | Faith’s PD | Decreased | Unweighted UniFrac | Significantly different |
| Kang DW 2017 | Faith’s PD | Decreased | NA | NS |
|  | Observed species | Decreased |  |  |
| Kong X 2019 | Shannon | NS | Weighted UniFrac  Unweighted UniFrac  Jaccard  Bray-Curtis | NS  NS  NS  NS |
|  | Faith’s PD | NS |  |  |
|  | Simpson | NS |  |  |
| Kushak RI 2017 | Chao1 | NS | Weighted UniFrac | NS |
|  | Shannon | NS | Unweighted UniFrac | NS |
| Li N 2019 | Faith’s PD | Increased | Unweighted UniFrac | Significantly different |
|  | ACE | Increased |  |  |
|  | Shannon | NS |  |  |
| Liu S 2019 | Shannon | Decreased | Weighted UniFrac | Significantly different |
|  | Sobs | NS |  |  |
|  | Chao | NS |  |  |
|  | ACE | NS |  |  |
| Nirmalkar K 2022 | Shannon | NS | Jaccard | NS |
|  |  |  | Bray–Curtis | NS |
| Niu M 2019 | Shannon | Increased | NA | NS |
|  | Simpson | NS |  |  |
| Pulikkan J 2018 | Observed species | NS | Unweighted UniFrac | Significantly different |
|  | Shannon | NS |  |  |
|  | Faith’s PD | NS |  |  |
| Sun H 2019 | Chao1 | NS | NA | NS |
|  | Observed species | NS |  |  |
|  | Shannon | NS |  |  |
| Tomova A 2020 | Chao1 | Increased | NA | NS |
|  | Observed species | Increased |  |  |
| Tong C 2022 | Shannon | NS | NA | NS |
|  | Richness | NS |  |  |
| Vernocchi P 2022 | Chao1 | Decreased | Weighted UniFrac  Unweighted UniFrac  Bray-Curtis | Significantly different  Significantly different  Significantly different |
|  | Observed species | Decreased |  |  |
|  | Simpson | Decreased |  |  |
|  | Good’s coverage | NS |  |  |
|  | Shannon | NS |  |  |
| Wan Y 2022 | Chao1 | Increased | NA | NS |
| Wang M 2019 | Chao1 | Decreased | Bray-Curtis | Significantly different |
| Wang Y 2020 | Simpson | Increased | Unweighted UniFrac | Significantly different |
|  | Shannon | Increased |  |  |
|  | Observed species | Increased |  |  |
|  | Chao1 | Increased |  |  |
| Williams BL 2011 | Shannon | NS | NA | NS |
| Wong OWH 2022 | Chao1 | Decreased | Weighted UniFrac  Unweighted UniFrac  Bray-Curtis | Significantly different  Significantly different  Significantly different |
|  | Faith’s PD | Decreased |  |  |
| Xie X 2022 | Shannon | Decreased | Weighted UniFrac  Unweighted UniFrac  Bray-Curtis | NS  NS  NS |
|  | Chao1 | NS |  |  |
|  | ACE | NS |  |  |
|  | Simpson | NS |  |  |
| Ye F 2021 | Observed species | Increased | Weighted UniFrac | Significantly different |
|  | Shannon | Increased |  |  |
|  | Simpson | Increased |  |  |
|  | Chao1 | NS |  |  |
| Yu R 2022 | Chao1 | Increased | Weighted UniFrac | Significantly different |
|  | Shannon | Increased | Unweighted UniFrac | Significantly different |
|  | Simpson | Increased | Bray-Curtis | Significantly different |
|  | Pielou’s eveness | Increased | Jaccard | Significantly different |
| Zhai Q 2019 | Chao1 | Increased | Bray-Curtis | Significantly different |
|  | Shannon | Increased |  |  |
| Zhang M 2018 | Shannon | NS | Bray-Curtis | Significantly different |
| Zhang Q 2021 | ACE | Increased | Weighted UniFrac | Significantly different |
|  | Chao1 | Increased |  |  |
|  | Shannon | Increased |  |  |
| Zhang Y 2020 | Faith’s PD | NS | Weighted UniFrac | Significantly different |
|  | Shannon | NS | Unweighted UniFrac | Significantly different |
|  | Observed species | NS | Bray-Curtis | Significantly different |
|  | Pielou’s evenness | NS | Jaccard | Significantly different |
| Zou R 2020 | ACE | Increased | Unweighted UniFrac | Significantly different |
|  | Chao | Increased |  |  |
|  | Shannon | Increased |  |  |
|  | Simpson | NS |  |  |
|  | Good’s coverage | NS |  |  |

Abbreviations: NA, not applicated; NS, no significant difference

**Figure S1.** Microbial lineage of differentially abundant taxa reported by 3 or more studies.

**
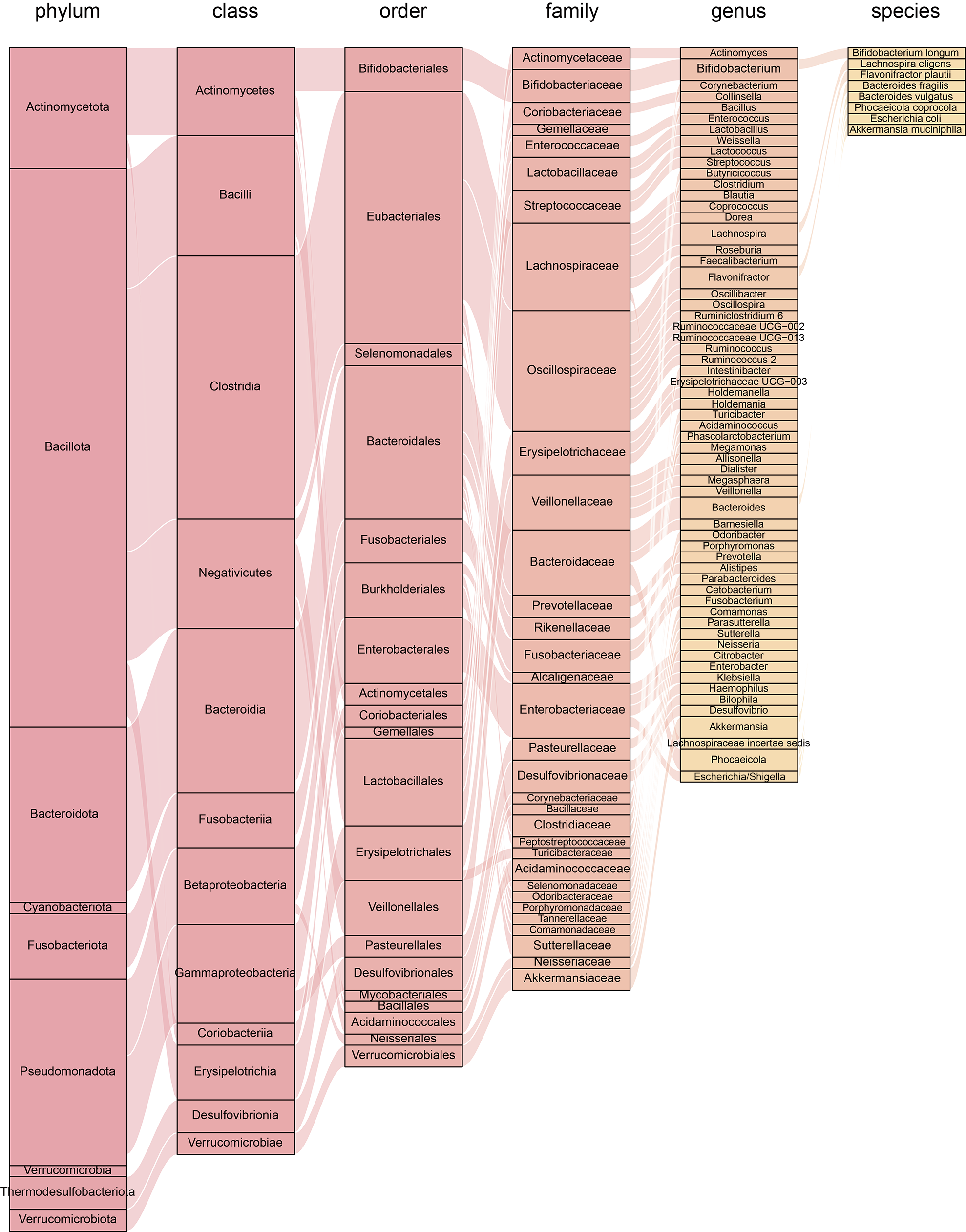
**

**Figure S2.** Bar plots of each microbiota that was consistently reported by ≥ 2 studies in ASD patients in ASD patients with gastrointestinal symptoms group (A), American group (B) and Chinese group (C). The vote counting statistic for each microbiota is represented by orange and blue bars. An asterisk (*) represents the difference was statistically significant.

**
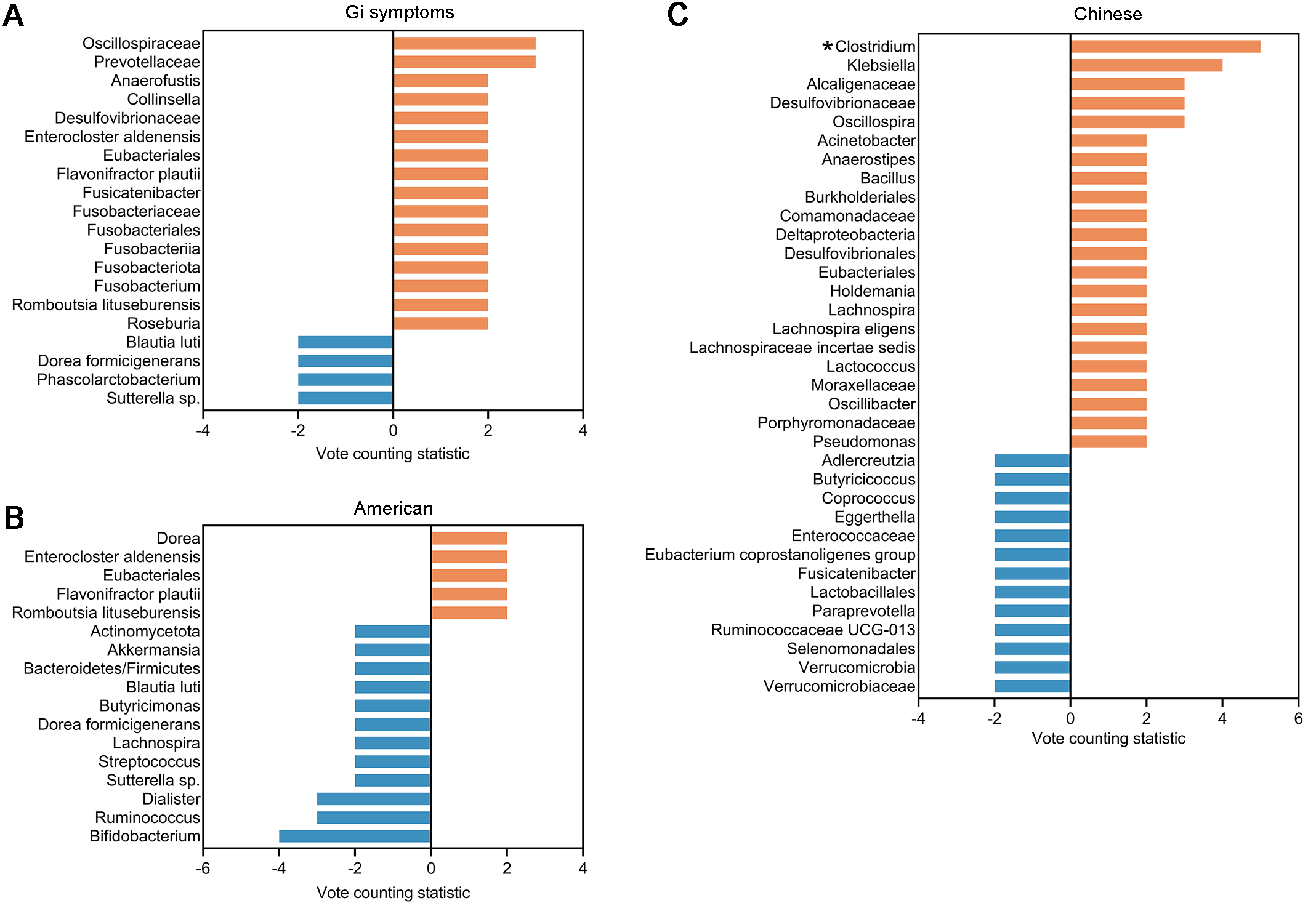
**

**Figure S3.** Bar plots of each microbiota that was consistently reported by ≥ 2 studies in 16S rRNA group at phylum (A), class (B), order (C), family (D), genus (E), species (F) and no rank (G) levels. The vote counting statistic for each microbiota is represented by orange and blue bars. An asterisk (*) represents the difference was statistically significant.

**
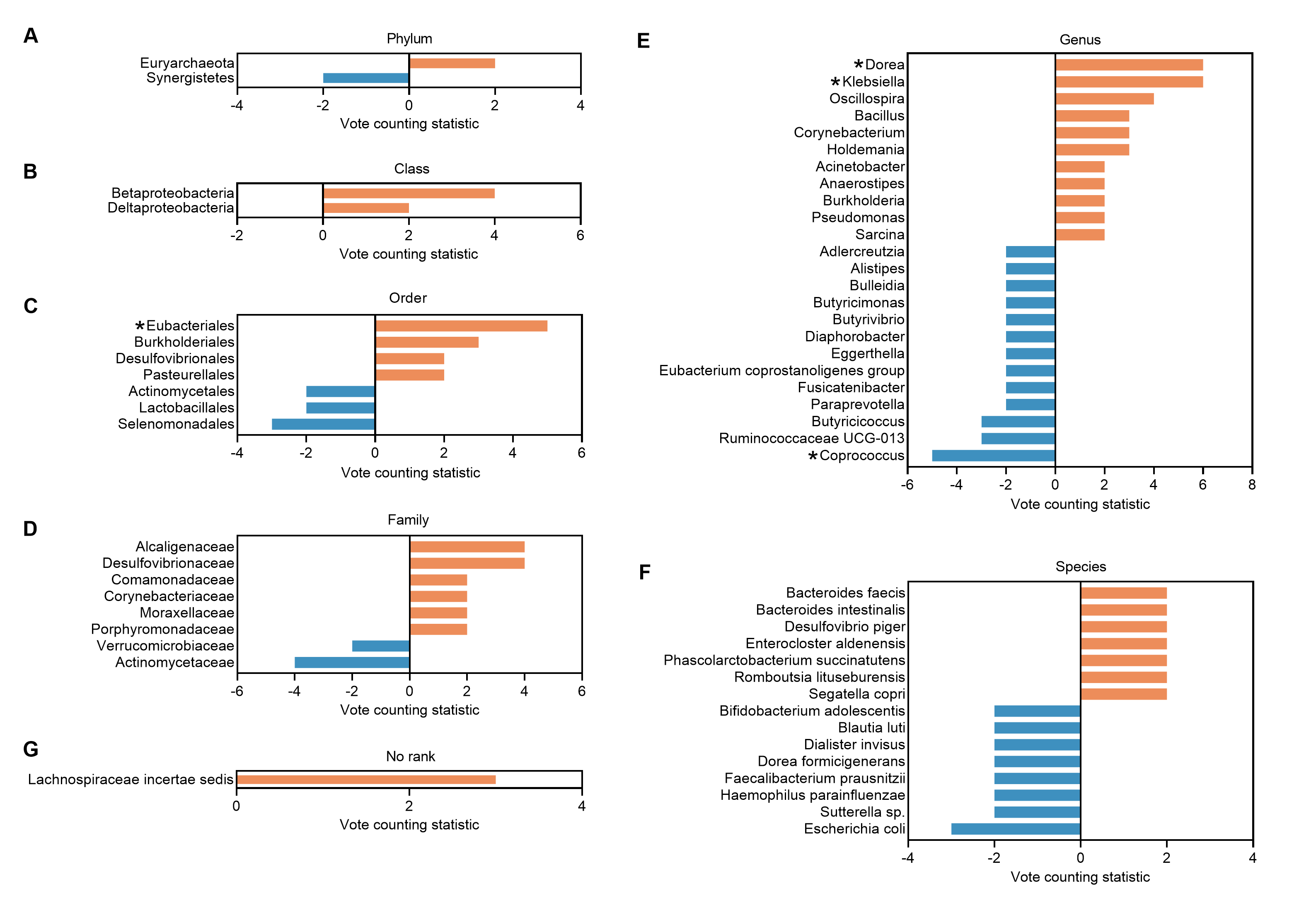
**

**Figure S4.** Volcano plots of differentially abundant taxa at 16S rRNA group that was reported by 3 or more studies. The x-axis indicates the vote counting statistic, while the y-axis shows the −log10 (*P*-value). The color of each plot represents the variation of the taxa; orange represents up-regulation, blue represents down-regulation and gray represents the variation that is not significant.


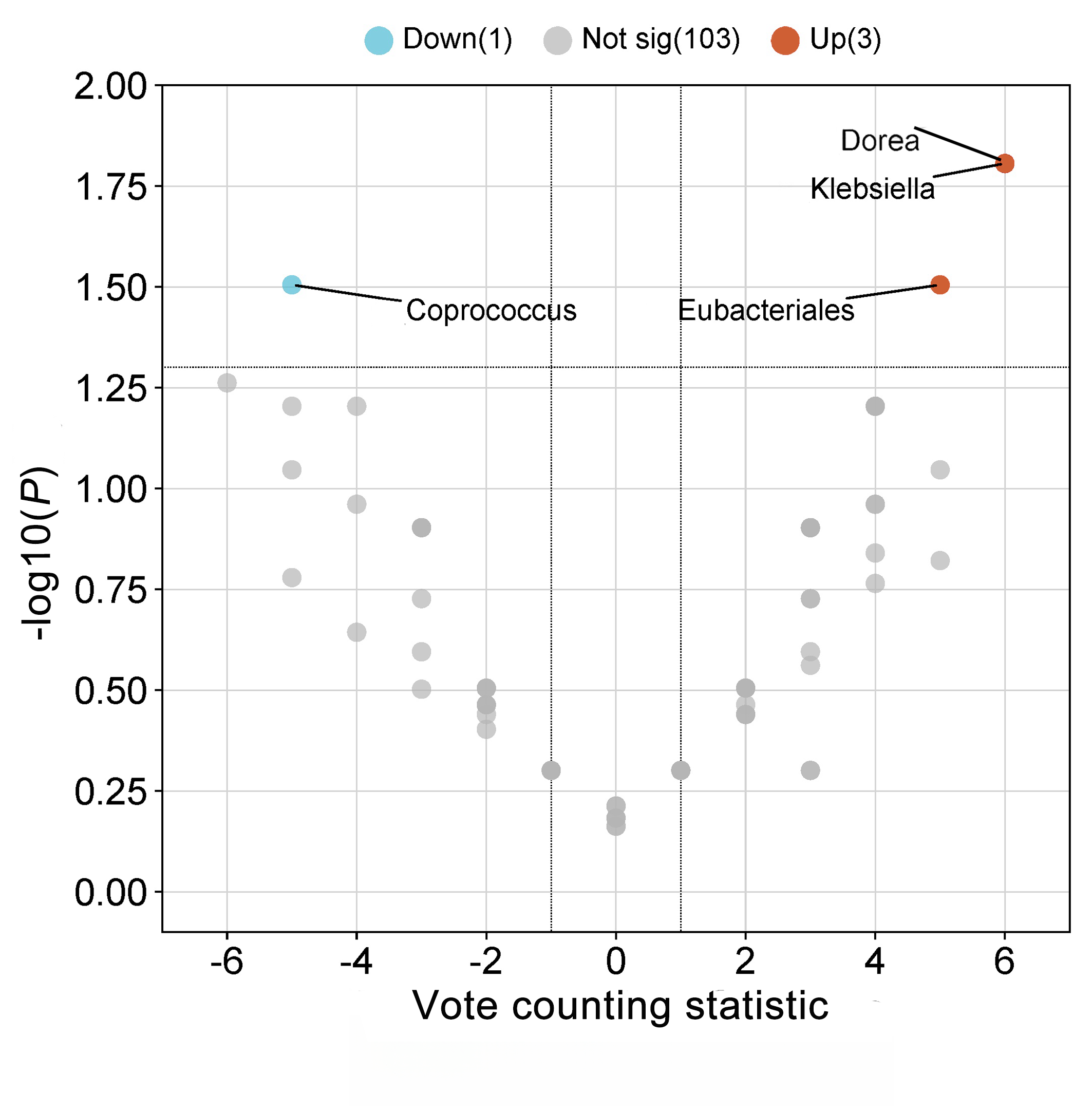

Supplement: Supplementary file 2 [file Data_Sheet_1.docx]
